# Quantitative polymerase chain reaction from malaria rapid diagnostic tests to detect *Borrelia crocidurae* in Mali

**DOI:** 10.1371/journal.pntd.0014033

**Published:** 2026-08-10

**Authors:** Pascal Dembélé, Adama Zan Diarra, Papa Mouhamadou Gaye, Armel Joseph Agokeng Dongmo, Li Bing, Mahamadou Ali Thera, Stéphane Ranque

**Affiliations:** 1 Institut Hospitalo-Universitaire Méditerranée Infection (IHU), Aix Marseille University, Marseille, France; 2 Aix-Marseille University, SSA, AP-HM, UMR D, Risques Infectieux, Microorganismes Emergents (RITMES), Marseille, France; 3 Programme National de Lutte Contre le Paludisme (PNLP), Bamako, Mali; 4 IRD, EMR Maladies Infectieuses, négligées et Emergentes au Sud (MINES), Dakar, Sénégal; 5 Department of Ophthalmology, Affiliated Hospital of Gui Zhou Medical University, Guiyang, China; 6 Malaria Research and Training Center (MRTC), FMOS-FAPH, Mali-NIAID-ICER, Université des Sciences, des Techniques et des Technologies de Bamako, Bamako, Mali; Yale University, UNITED STATES OF AMERICA

## Abstract

**Background:**

Tick-borne relapsing fever (TBRF) is caused by *Borrelia* species transmitted to humans by soft ticks of the genus *Ornithodoros*. Very little is currently known about the morbidity of this disease in Mali, despite the risk of human co-infection with malaria. The lack of appropriate diagnostic services or technical expertise to differentiate suspected malaria from other causes of febrile illness of unknown origin means that this tick-borne disease remains neglected and under-diagnosed in febrile patients in Mali. Our study investigated the detection of *Borrelia crocidurae* DNA in febrile patients in Mali from malaria rapid diagnostic tests (RDTs) for *Plasmodium falciparum*.

**Methodology/Principal Findings:**

Between June and December 2021, negative and positive malaria RDTs were collected from 41 health centers in the nine regions of Mali. Both qPCR and standard PCR were used to detect the presence of *B. crocidurae* DNA.

**Results:**

Of the 1,496, malaria RDTs tested, *B. crocidurae* DNA was detected in 9 DNA extracts from malaria-negative RDTs. All of these were collected in the region of Kayes, where the prevalence rate was 5% (9/180) of the malaria RDTs*.*

**Conclusions/significance:**

This study demonstrates that tick-borne relapsing fever is an under-diagnosed condition in Mali.

## Author summary

RDTs (rapid diagnostic tests) are primarily used to detect *Plasmodium falciparum*, and other *Plasmodium* species, in patients with a fever. RDTs have been shown to reliably provide bacterial DNA for investigating acute undifferentiated febrile illnesses and the pathogens responsible for neglected tropical diseases transmitted by arthropod-borne parasites. Tick-borne relapsing fever (TBRF) is an under-recognized disease in Mali that is often mistaken for malaria due to the similarity of the symptoms and the lack of adequate diagnostic tools. We used DNA extracted from malaria RDTs collected in different regions of Mali to screen for the bacterium *Borrelia crocidurae*, which causes TBRF. *Borrelia crocidurae* DNA was identified in nine malaria-negative RDTs, which were collected exclusively in the Kayes region. The methodological approach of this study made it possible to estimate the prevalence of *Borrelia* infection in western Mali, representing a significant breakthrough in the mapping of human cases of borreliosis. The presence of positive *B. crocidurae* DNA extracts from malaria RDTs confirmed the geographical distribution of borreliosis in western Mali. Although it is rarely diagnosed, we believe that TBRF is a common cause of morbidity in Mali’s endemic areas.

## 1. Introduction

Tick-borne relapsing fever (TBRF) is a neglected febrile infection caused by spirochetes of the genus *Borrelia* and transmitted to humans by soft ticks of the genus *Ornithodoros* [1,2]. Wild rodents and insectivores are common reservoir hosts. They are hosting several spirochetes of the *Borrelia* species (*B. crocidurae, and B. hispanica*), which are endemic in subtropical regions worldwide [3,4]. This acute febrile illness causes multiple recurrences of nonspecific signs and symptoms, including fever, headache, hepatomegaly, splenomegaly, anemia, myalgia, and arthralgia. These symptoms are similar to those of malaria [5,6]. The first clinical cases of TBRF have long been described as a major cause of morbidity and mortality in many parts of Africa [7]. In West Africa, *B. crocidurae* is recognized as the only causative agent of TBRF. Rural populations living in traditional dwellings made of mud and sleeping on the floor are the most vulnerable [8–10]. Studies have shown that the incidence of TBRF in West Africa is high, accounting for 13% of febrile illnesses treated in rural health facilities [2].In Tanzania, particular attention is currently being paid to TBRF, which is one of the ten leading causes of death in children under 5 year of age [11].

The conventional diagnosis of TBRF relies on the microscopic detection of the spirochete in blood smears sampled during the acute febrile phase. However, TBRF is underdiagnosed in most disease-endemic areas, where blood smears are only screened for malaria parasites [12]. On the other hand, PCR is the diagnostic tools because of their high sensitivity and specificity [13].The lack of diagnostic tools in Africa has been identified as a major gap in healthcare delivery on the continent [14]. In Mali, for example, studies have reported the presence of *B. crocidurae* in tick vectors and small mammal rodents, which are the reservoirs [15–17]. It has been shown that West African tick-borne relapsing fever (TBRF) is widespread, especially in the Saharan, Sahelian and Sudano-Sahelian regions, where average annual precipitation is between 50 and 250 mm, 250 and 500 mm and 500 and 750 mm respectively [18]. The persistence of drought in sub-Saharan countries potentially contributes to the spread of *Ornithodoros sonrai* vector responsible for transmitting *B. crocidurae* [19,20]. Most studies on TBRF in West Africa have been carried out in Senegal [12,21,22], where the TBRF vector ticks are geographically distributed over the northern two-thirds of the country, north of the 750 mm isohyet, and the southern limit of the vector tick corresponds approximately to latitude 13°40’N [18].

However, TBRF remains the second major challenge in many African countries where malaria is endemic, including Mali, where microscopic examination of blood smears remains the gold standard for malaria diagnosis [23]. Because of the rarity of appropriate diagnostic services, such as molecular biology laboratories, it is clear that TBRF cannot be diagnosed quickly and reliably [24]. Indeed, when misdiagnosed, many cases of TBRF will be considered treatment-resistant malaria, as studies in Togo have shown, where febrile patients were often misdiagnosed as malaria [5]. Malaria (RDTs) are the main form of medical diagnosis used for diagnosing malaria in low-income countries [25].They can significantly improve the quality of care for malaria, particularly in remote areas with limited access to high-quality microscopy services [26]. However, several studies have pointed to their usefulness as a potential source of DNA for molecular analysis [6,27–29]. DNA extracts from RDTs for *P. falciparum* was used to determine the prevalence of TBRF among patient with acute undifferentiated febrile illness in Mali.

## 2. Materials and methods

Ethical statement: The use of malaria RDTs in this study raises no ethical concerns, as they had been previously used for routine medical diagnosis at healthcare facilities and they were destined for destruction. The malaria RDTs were de-identified to ensure patient confidentiality and no data regarding patient identity or socio-demographic characteristics were collected or used in the study. In addition, regulatory approval for their use was obtained from the competent authority of the National Malaria Control Program (PNLP), a service under the General Secretariat of the Ministry of Health and Social Development, under reference number 064/MSDS-SG/PNLP.

### 2.1. Study areas

**Fig 1:**
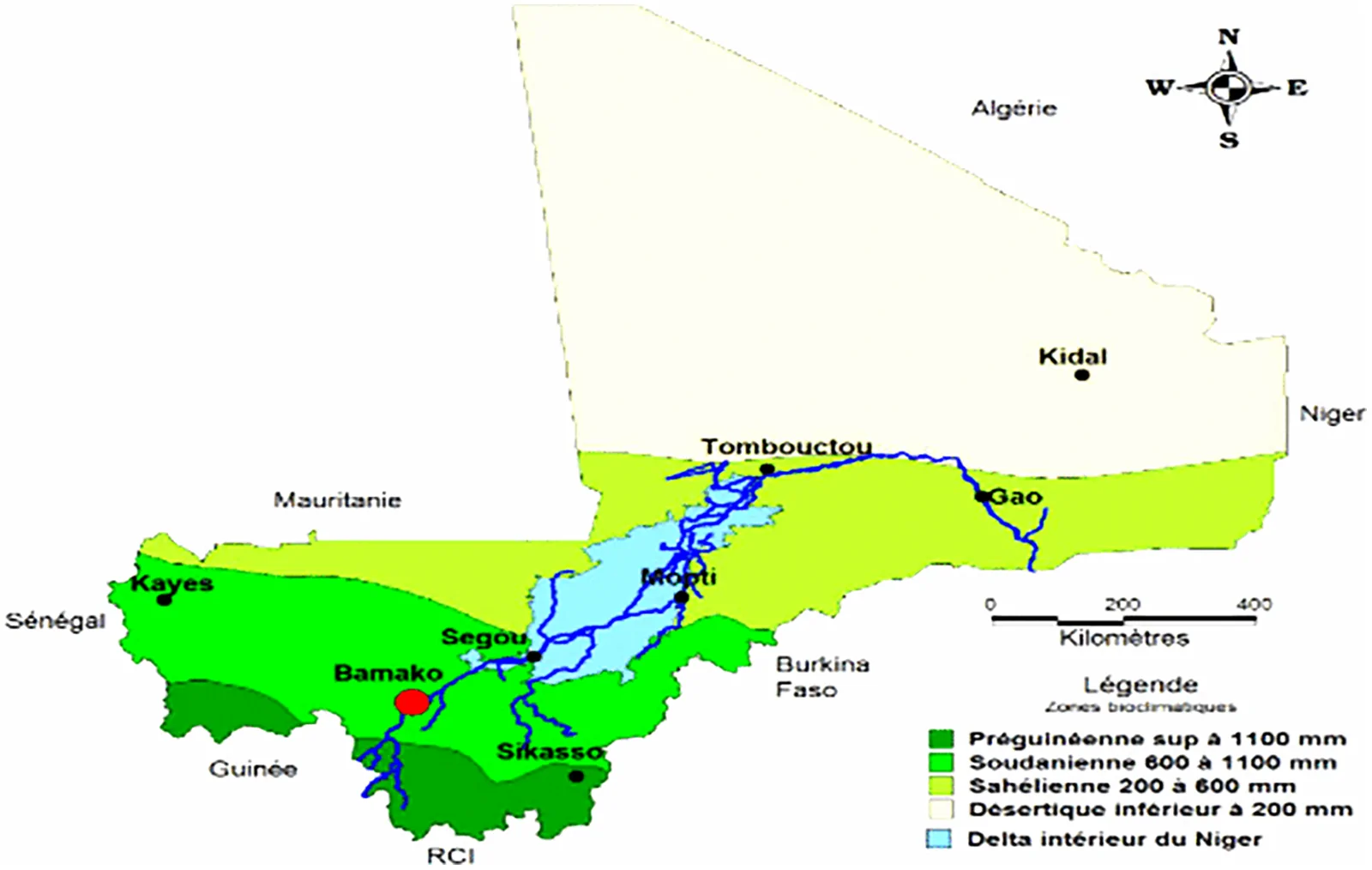
Map of Mali showing climatic zones. The base layer for the map was sourced from: https://data.humdata.org/dataset/mali-formation-sanitaires.

Mali is a landlocked country in West Africa located between the 10- and 25-degree north latitudes and between the 4 - and 12 degree west longitudes. It covers a 1,241,238 km2 area. There are 3 climatic zones in Mali that extend from south to north: the Sudano-Guinean zone (Fig 1), which covers 25% of the territory and has a rainfall of approximately 1300–1500 mm per year; the Sahelian zone, which covers 50% of the territory and receives rainfall of 200–800 mm per year; and the Saharan desert zone, which represents 25% of the territory. This zone is marked by irregular rainfall, often less than 200 mm per year. Malaria RDTs were collected from 41 health centers in 9 regions of Mali, namely: Kayes, Koulikoro, Sikasso, Ségou, Mopti, Timbuktu, Gao, Kidal and Menaka, from June to December 2021.

All *P. falciparum* malaria screening RDTs were stored at room temperature from the time of collection until DNA extraction at the laboratory of the Institute for Research on Mediterranean Infections in Marseille, France. The time elapsed between testing and DNA extraction can be estimated at one year.

### 2.2. Study design

#### 2.2.1. Rapid Diagnostic Test (RDT).

In Mali, the purchase and distribution of rapid diagnostic tests is an essential part of the master plan for the supply and distribution of essential medicines (SDADME). To control consumption needs and guarantee their availability and quality, they are managed similarly to other medicines, by using the same management tools. Decision No. 2011–774/ Ministry of Health General Secretariat (MS-SG) of 11 July 2011 made their application mandatory. Its objective is to ensure the correct supply of health products to the population by the Popular Pharmacy of Mali (PPM), which is the State’s preferred tool for the supply, storage and distribution of health products through a State-PPM contract plan. This system is supplemented by the private sector through the Private Import and Wholesale Establishments (EPIWG) of approved private suppliers of pharmaceutical products. The malaria RDTs for *P. falciparum* used in our study sites were the SD Bioline Pf Ag (Standard Diagnostics, Inc, 05FK50), Adv Dx Malaria Pf Ag HRP2 (J. Mitra & Co. Pvt. Ltd, IR016025), which both detect the *P. falciparum* specific Histidin-Rich Protein 2; and First Response Malaria Ag pLDH/HRP2 (Premier Medical Corporation Ltd, I16FRC25), which also detects the Lactate Dehydrogenase (pLDH) of *Plasmodium* species. Malaria RDTs were performed by health workers to reach a diagnosis of malaria in febrile outpatients presenting at health facilities (Table 1). They were selected proportionally to the different collection sites in the region before including positive and negative malaria RDTs*.*

**Table 1:** Detail of the total number and the prevalence of positive and negative Malaria Rapid Diagnostic Tests in each health center.

Region	Health district	RDTs collection in health centers	Geographic Coordinate from Health centers		Result		Total
			Latitude	longitude	Positive N (%)	Negative N (%)	
Kayes	Diéma	Torodo	14.51132°	-8.84285°	6 (2.2)	272 (97.8)	278
		Lakamané	14,50616°	-9.9070°	3 (3.4)	89 (96.7)	92
		Lattakaf	14.53213°	-9.72795°	0 (0.0)	47 (100)	47
		Débomassassi	14.58019°	-9.36827°	0 (0.0)	48 (100)	48
		Koungo	14.91472°	-9.36405°	3 (75.0)	1 (25.0)	4
		Lambidou	14,31315°	-9,5512°	0 (0.0)	13 (100)	13
		Diancounté Camara	14,5450°	-9,5137°	3 (6.4)	44 (93.6)	47
	Yélimané	Kodié	15,201988°	-10,5333°	12 (19.0)	51(81.0)	63
		C.S.Réf. Yelimané	15,118000°	-10.5720°	6 (4,1)	139 (95.9)	145
		Dogofry	15,0125°	-10.94056°	0 (0.0)	23 (100.0)	23
		Bandiougoula	15,24825°	-1050323°	0 (0.0)	11 (100.0)	11
*sub total*					*33 (4.3)*	*738 (95.7)*	*771*
Koulikoro	Nara	Bagoïni	15,17316°	-8.63241°	0 (0.0)	50 (100.0)	50
		Mourdiah	14,4498°	-74754°	5 (7.4)	68 (100.0)	73
		Kassakaré	15,3672°	-8,2372°	4 (12.1)	29 (87.9)	33
		Alasso	14,924°	-8.023°	7 (20.0)	28 (80.0)	35
		Tiapato	15,17254°	-7.78638°	3 (8.8)	31 (91.2)	34
	Kangaba	Naréna	12,2039°	-8,6186°	2 (1.6)	126 (98.4)	128
		CSCOM Central	11,9191°	-8,4074°	14 (28.0)	36 (72.0)	50
		Séléfougou	11,7186°	-8,3199	0 (0.0)	30 (100.0)	30
*sub total*					*35 (8.1)*	*398 (91.9)*	*433*
Sikasso	Kadiolo	CSCOM central	10,55404°	-5.75929°	110 (59.5)	75 (40.5)	185
		Kebeni	10,25699°	-6.12128°	12 (16.4)	69 (85.2)	81
		Zégoua	10,4828°	-5.65314°	28 (38.9)	44 (61.1)	72
*sub total*					*150 (44.4)*	*188 (55.6)*	*338*
Segou	Barouéli	Cscom Central	13,0749°	-6.5717°	5 (41.7)	7 (58.3)	12
		Dioforogo	13,10546°	-6.49597°	13 (65.0)	7 (35.0)	20
		Tamani	13,33843°	-6.83488°	7 (35.0)	13 (65.0)	20
		NGara	13,30992°	-6.50518°	16 (80.0)	4 (20.0)	20
		Tigui	13,05936°	-6.6448°	20 (100.0)	0 (0.0)	20
		Bananido	12,87475°	-6.83998°	7 (50.0)	7 (50.0)	14
		N’Gossola	13,04784°	-5.11189°	5 (31.2)	11(68.8)	16
		Nianzana	13,09344°	-6.99751°	20 (66.7)	10 (33.3)	30
		Yerebougou	13,03752°	-6.57322°	16 (80.0)	4 (20.0)	20
		C.S.Réf. Segou	13,4415°	-6.2681°	8 (6.2)	122 (93.8)	130
		Ndjila	12,96797°	-6.95901°	10 (50.0)	10 (50.0)	20
*sub total*					*127 (39.4)*	*195 (60.6)*	*322*
Mopti	Mopti	Soufouroulaye	14,4074°	-4.08268°	79 (34.3)	151 (65.7)	230
		Fatoma	14,61356°	-4.05993°	0 (0.0)	35 (100.0)	35
		Sévaré II	14,5224°	-4.0921	136 (91.9)	12 (8.1)	148
*sub total*					*215 (52.1)*	*198 (47.9)*	*413*
Tombouctou		Gourma Rharous	16,87767°	-1.92535°	379 (65.7)	198 (34.3)	577
Gao	Gao	C.S.Réf. d’Ansongo	16,2552°	-0,0289	30 (13.4)	194 (86.6)	224
Menaka	Menaka	Menaka	15.9182°	2.4022°	21 (36.8)	36 (63.2)	57
*sub total*					21 (36.8)	36 (63.2)	57
Kidal	Kidal	CSCOM d’Aliou	18.42667°	1.41586°	9 (14.3)	54 (85.7)	63
		C.S.Réf. de Kidal	18,4446°	1,4122	20 (14.3)	120 (85.7)	140
*sub total*					*29 (14.3)*	*174 (85.7)*	*203*
**Total**					**1019 (30.5)**	**2319 (69.5)**	**3338**

3338 malaria RDTs were collected at the various study sites, of which 69.5% were negative for malaria.

#### 2.2.2. Inclusion and exclusion criteria.

We included malaria RDTs that were either negative for *P. falciparum*, with a single band visible in the control window “C”, or positive for *P. falciparum*, with a band both in the “C” control and in the “T” test windows. Malaria RDTs for *P. falciparum* with no band in the “C” control window were excluded.

#### 2.2.3. DNA extraction.

The selected malaria RDTs were opened under a Type 2 Microbiological Safety Station (MSS) using a dissecting needle (Lanceolee Models LT2304, Pakistan), dissecting forceps, and a pair of scissors. The nitrocellulose tape was removed from the plastic cassette and then cut into small 3 × 3 mm pieces using scissors for each sample [6].The scissors were decontaminated between each sample with 70° ethanol. The cut nitrocellulose samples were then introduced into 1.5mL collection tubes for incubation at room temperature.

***Borrelia* spp. DNA detection by real-time PCR:** DNA extraction for *Borrelia* detection from malaria RDTs was performed as previously described [30].The DNA templates were subjected to real-time polymerase chain reaction (qPCR) using primers and probes targeting the internal transcribed spacer (ITS) region of the *Borrelia* spp. rRNA gene. The primer sequences used were Bor_ITS4_F 5’-GGCTTCGGGTCTACCACATCTA-3’ and Bor_ITS4_R 5’-CCGGGAGGGGAGTGAAATAG-3’; and the Bor_ITS4_ P probe sequence was 6FAM-TGCAAAAGGCACGCCATCACC TAMRA [16].The samples that tested positive for *Borrelia* spp. by ITS PCR were subjected to a second qPCR targeting the glpQ gene specific for *B. crocidurae* using the primers (croci_glpQ_F5’-CCTTGGATACCCCAAATCATC-3’ and croci_glpQ_R5’-GGCAATGCATCAATTCTAAAC-3’) and the *B. croci*_glpQ_P probe (6FAM-ATGGACAAATGACAGGTCTTAC -MGB) [31,32]. The qPCR mix composition and reaction steps were the same as those previously described by Keita et al [33]. To validate our results, each PCR analysis was performed with negative controls and a positive control derived from DNA of *B. crocidurae* strains grown in the laboratory. Samples were considered positive if the qPCR cycle threshold (Ct) was less than 36 [32].

**Fig 2:**
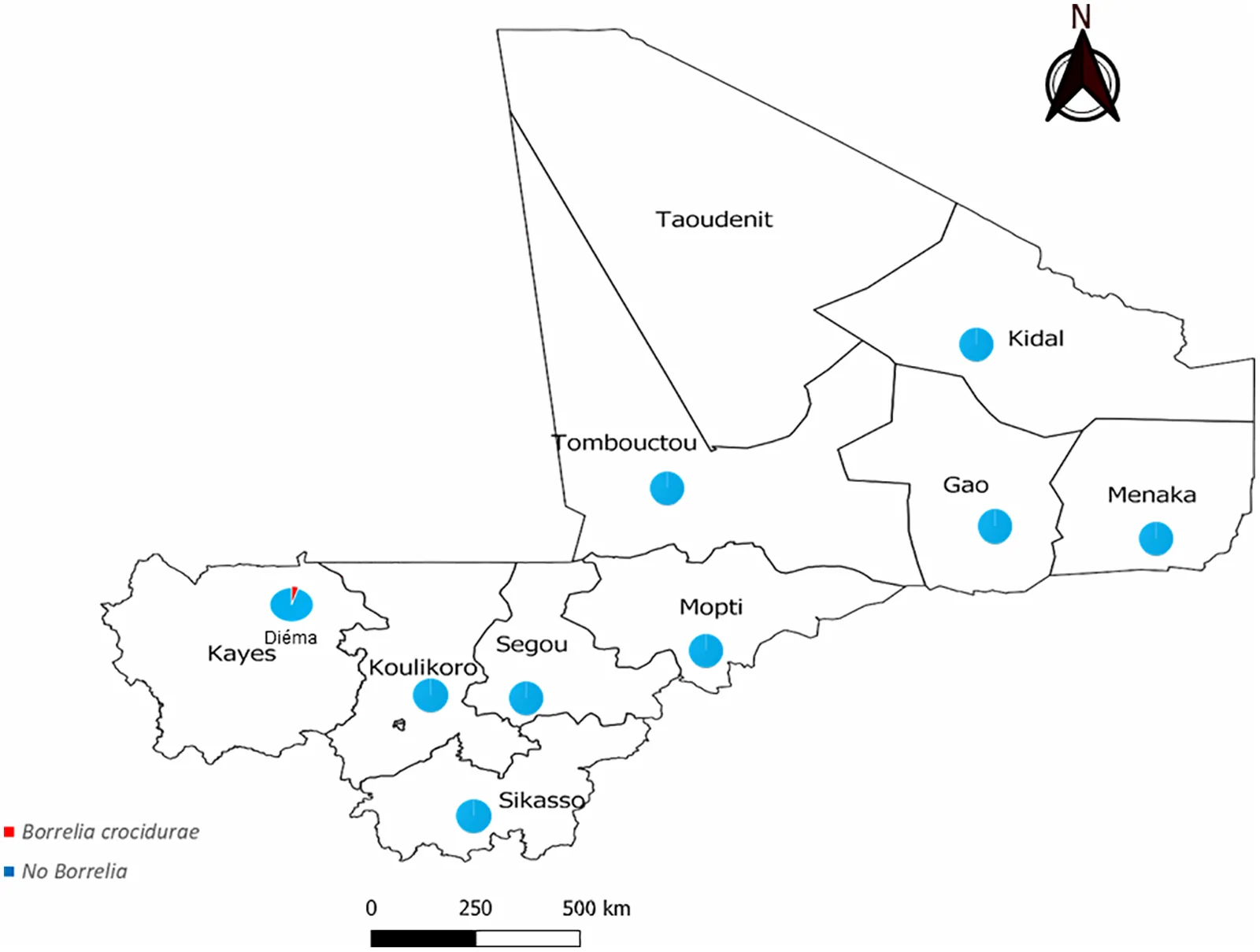
Map of Mali showing the localities where malaria RDTs for *P. falciparum* were collected in health centers and results of molecular analysis. The base layer for the map was sourced from: https://data.humdata.org/dataset/mali-formation-sanitaires.

**DNA sequencing:** All samples positive for the two genes by qPCR were subjected to standard PCR and sequencing. Standard PCR amplification targeting the Flagellin *flaB* gene (B_F1 (5’-TAATACGTCAGCCATAAATGC-3’ and B_R1 (5’-GCTCTTTGATCAGTTATCATTC-3’) [12] was performed using a thermocycler (Applied Biosystems, Foster City, CA, USA). The composition of the mixture and the standard PCR program and sequence were the same as those previously described by Rahal et al [34]. The amplified products were purified using a Macherey Nagel plate (NucleoFast 96 PCR, Düren, Germany) and sequenced using the same primers (B_F1 and B_R1). Sequencing was performed using the same primers as for PCR with BigDye Terminator v1.1, v3.15 × sequencing buffer (Applied Biosystems, Warrington, UK) and performed on an ABI 3100 automated sequencer (Applied Biosystems). The resulting sequences were assembled and processed using ChromasPro (www.technelysium.com.au/chromas.html) [35] and aligned using BioEdit software (http://www.mbio.ncsu.edu/BioEdit/bioedit.html). The corrected sequences were compared with existing sequences in GenBank (http://blast.ncbi.nlm.nih.gov/Blast.cgi) using basic local alignment search tool (BLAST) analysis. The sequences of samples positive for *Borrelia crocidurae* by qPCR are shown in Fig 2. Phylogenetic trees were constructed using the maximum likelihood method with model selection, by using the Molecular Evolutionary Genetics Analysis (MEGA) v7.0.26 software (Tamura K, 1993). The statistical support of the internal branches of the trees was assessed by bootstrapping with 1000 iterations.

## 3. Results

### 3.1. Collection of malaria RDTs in the targeted health centers

During the study period, 3,338 malaria RDT for *P. f* were collected from 41 Health facilities in the 9 regions of Mali, of which 1,019 (30.5%) were positive (Table 1). In Table 1, the number of malaria RDTs for *P. falciparum* and the prevalence of positive malaria RDTs for *P. falciparum* is detailed in each study site. Overall, 3,338 malaria RDT were collected, of which (69.5) tested malaria negative.

We then randomly selected 30 positive and 150 negative malaria RDTs in each administrative region for further qPCR analyses, except in two regions, namely Menaka and Kidal, where all positive tests (21 and 29 for Menaka and Kidal, respectively) and all negative tests (n = 36) in Menaka were included. Among the 1,502 malaria RDTs selected, 6 were excluded as invalid, and *Plasmodium* spp. detection was positive in 260, and negative in 1,236.

Firstly all 1,496 malaria RDTs were further analyzed using a *Plasmodium* spp. Among them, 258 had a positive *Plasmodium* spp. qPCR. Among these 258 *Plasmodium*-positive samples, 251 (96.9%) were positive for *P. falciparum***,** 5 (1.9%) for *P. vivax* and 2 (0.8%) for *P. malariae*. We detected two cases of mixed infections, where *P. falciparum* was combined either with *P. vivax* or *P. malariae*, in Kidal and Koulikoro, respectively.

### 3.2. qPCR detection of *Plasmodium* spp. DNA in malaria RDTs

Of the 1,496 malaria RDTs analyzed, *Plasmodium* spp. DNA was detected by qPCR in 260 samples. The *Borrelia* spp. *ITS4* qPCR tested positive in only 9/1,496 samples (0.6%). No case of *B. crocidurae-Plasmodium* co-infection were found*.* The 9 *Borrelia* spp. ITS4 qPCR positive samples were then tested using the *glpQ* qPCR, specific for *B. crocidurae*. *B. crocidurae* DNA was detected in all ITS4 qPCR-positive samples, corresponding to 5% (9/180) of the randomly selected malaria RDTs collected in the Kayes region.

Sequencing of the central region of the *flaB* gene yielded nine partial sequences ranging in length from 322 to 346 bp. Comparison of the central region of all partial *flaB* gene sequences of *Borrelia* obtained in this study with sequences in GenBank revealed 99–100% coverage and 100% identity with *B. crocidurae* sequences (JX119098, GU357619, and JX29291), detected, either in human blood in Senegal, or in *O. sonrai* in Mauritania, and in Mali.

**Fig 3:**
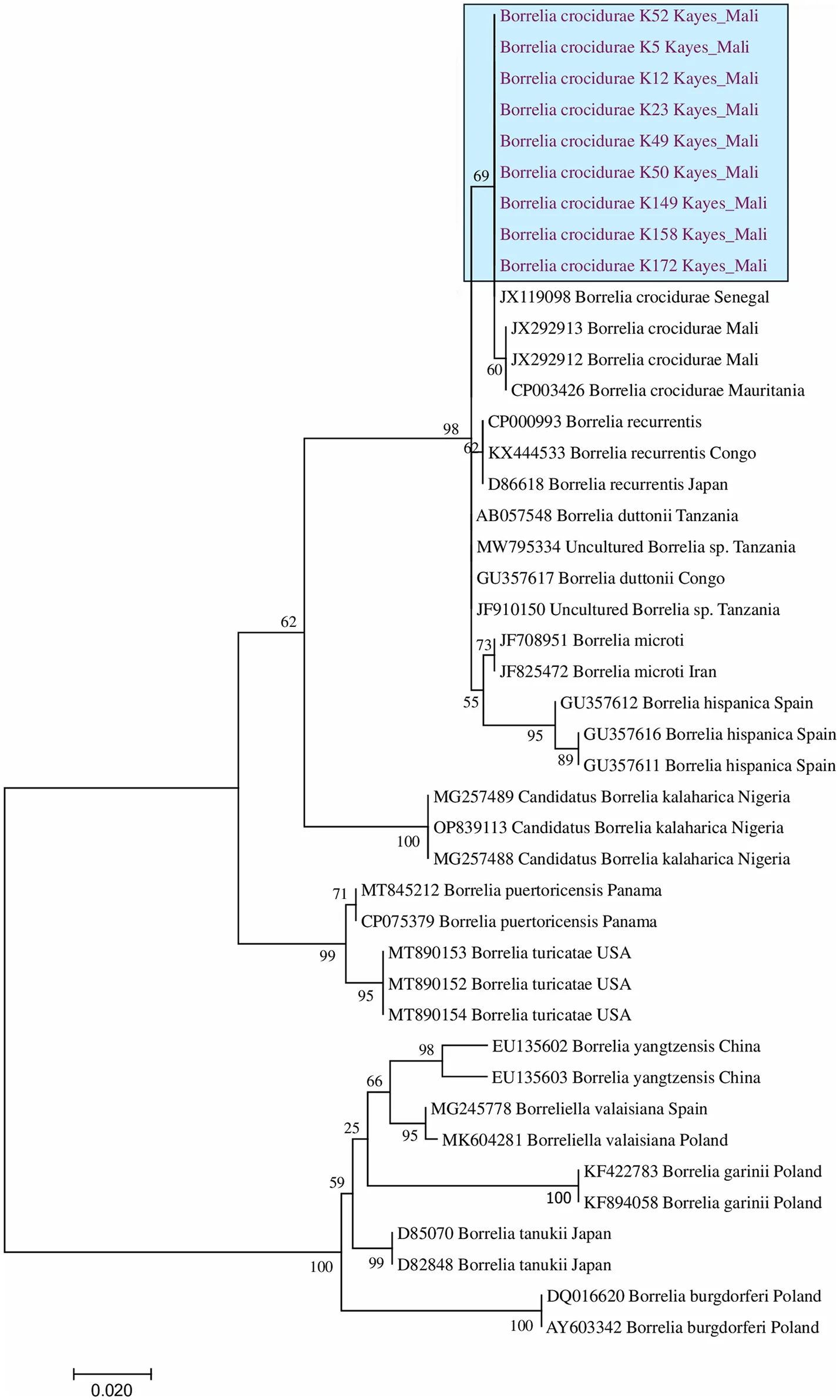
Phylogenetic tree constructed using the maximum likelihood method with 1,000 bootstrap replicates, based on partial sequences of the flagellin gene (flaB) from *Borrelia crocidurae* generated from RDT samples in this study (blue box). Sequences from several *Borrelia* species obtained from GenBank were included in the phylogenetic tree for comparison.

The phylogenetic tree (Fig 3) shows that our sequences are close to those already found in Mali, Mauritania and Senegal. The *flaB* gene sequences from this study are available in GenBank under the following accession numbers: PZ232043, PZ232044, PZ232045, PZ232046, PZ232047, PZ232048, PZ232049, PZ232050, and PZ232051.

Sequences of the *B. crocidurae flaB* gene found in *O. sonrai* ticks [17] and sequences from other *Borrelia* species were processed for comparison. The tree with the highest likelihood (-1092.19) is shown.

## 4. Discussion

In this study, we found a relatively high prevalence of 6% *B. crocidurae* in malaria RDTs in a rural health center in the Diema health district, which is located in the Kayes region bordering Senegal and Mauritania, two countries where *Borrelia* is endemic [2,3]. Phylogenetic analysis of the *flaB* gene showed that the *B. crocidurae* in our study were genetically close to those previously found in Senegal and Mali [15]. The results presented in this study confirm the involvement of *B. crocidurae* causing TBRF in non-malaria fever cases in Mali. Our main study limitations were that we were unable to visit all the health centers where malaria RDTs were collected due to the insecurity that has prevailed in the country since 2012. Good cooperation sometimes made it easier to obtain RDTs from certain health centers located in areas that are very difficult to access. It must also be acknowledged that storage conditions are not always optimal after the RDTs have been used. Dust, heat, and humidity could reduce the quality of samples.

Tick-borne relapsing fever (TBRF) is the most common vector tick-borne bacterial human disease in West Africa [10]. A similar study by Ndiaye *et al*. found a 7% prevalence of B. crocidurae in malaria RDTs collected in Senegal [7]. Between December 2007 and October 2011, a study conducted in 20 villages in southern Mali reported the presence of the tick vector *O. sonrai* in rodent burrows, with 17.3% of the collected ticks infected with *B. crocidurae* [15]. Most studies of TBRF in West Africa have been conducted in Senegal [12,21,36]. Our *B. crocidurae* prevalence found in Kayes (5%, 9/180) similar to that of Mediannikov *et al*. conducted in Senegal between June 2010 and October 2011, where 115 (7.3%) of 1,566 blood samples from febrile patients were positive for *B. crocidurae*. The highest proportion of positive samples was found in Niakhar with 19.1% (33/173) [21]. Patients aged 7–15 years were most affected: 13.5% (43/318). The proportion of *Borrelia*-positive samples was significantly higher in samples collected during the dry season (16.9%, 40/237) than during the rainy season (6.9%, 30/432) [21]. Several previous studies have demonstrated the influence of climate change on infectious diseases [37]. In December 2016, among febrile patients examined in Niakhar, Ngayokheme, Toucar and Diohine, cases of borreliosis accounted for 12% (94/800) of fever episodes, and all age groups were infected, with children and adolescents aged 8–14 and 22–28 years being most affected by the disease (16% and 18.4%) [36].The increase in the number of cases indicates that tick-borne relapsing fever (TBRF) poses a serious threat to public health in Senegal [36]. However, further studies are needed in neighboring Mali to determine the actual impact of TBRF on public health, as the present study detected only nine cases of *B. crocidurae* DNA carriage, all of which were found exclusively in the Kayes region in western Mali [36].

## 5. Conclusion

The surveillance system for vector-borne diseases such as borreliosis needs to be strengthened, and diagnostic tools for TBRF that are sensitive and much more specific for *B. crocidurae* need to be adopted to improve the management of this endemic zoonotic infection in Mali. Strengthening the skills of microscopists may be one way to improve the diagnosis of the disease in endemic rural areas from Mali and West African countries where qPCR diagnosis is unavailable.

## Supporting information

S1 Data *Borrelia crocidurae*-specific real-time PCR targeting the glpQ gene, performed on malaria rapid diagnostic tests samples collected in the Kayes region, Mali. The report details the 96-well plate layout, positive (T+) and negative (T−) controls, and crossing point (Cp/Ct) values for positive samples. Analyses were conducted using LightCycler 480 (LCS480) software.
(PDF)

S2 Data Real-time PCR targeting the internal transcribed spacer (ITS4) results for the screening of *Borrelia* spp. on malaria rapid diagnostic tests samples collected in the Kayes region, Mali. The report includes the 96-well plate layout, positive (T+) and negative (T−) controls, and crossing point (Cp/Ct) values for positive samples. Analyses were performed using LightCycler 480 (LCS480) software.
(PDF)

S1 Text *Borrelia crocidurae* flagellin B (flaB) gene sequences identified in malaria RDT samples from Mali, deposited in the NCBI/GenBank nucleotide database. (TXT)
