# Supplementary material for: Quantitative polymerase chain reaction from malaria rapid diagnostic tests to detect Borrelia crocidurae in Mali
Source: PLoS Negl Trop Dis. 2026 Aug 10;20(8):e0014033. doi: 10.1371/journal.pntd.0014033 (PMC13521451; doi:10.1371/journal.pntd.0014033)
Supplement: Supplementary file 1 [file pntd.0014033.s001.PDF]

Bor GLPQ Kayes du 31-07-23 Pascal

Experiment

Creation Date	08/01/2023 4:23:40 AM	Last Modified Date	08/01/2023 5:53:26 AM
Operator	System Admin	Owner	System Admin
Start Time	08/01/2023 4:28:25 AM	End Time	08/01/2023 5:42:16 AM
Run State	Completed	Software Version	LCS480 1.5.1.62
Macro		Macro Owner	
Macro Status			
Templates	Borrelia_Pascal	Plate ID	B9317037
Test ID		Lot ID	
Color Comp ID			
Run Notes			

Programs

Program Name	Program		
Cycles	1	Analysis Mode	None

Target (°C)	Acquisition Mode	Hold (hh:mm:ss)	Ramp Rate (°C/s)	Acquisitions (per °C)	Sec Target (°C)	Step size (°C)	Step Delay (cycles)
50	None	00:02:00	4.40		0	0	0

Program Name	Program		
Cycles	1	Analysis Mode	None

Target (°C)	Acquisition Mode	Hold (hh:mm:ss)	Ramp Rate (°C/s)	Acquisitions (per °C)	Sec Target (°C)	Step size (°C)	Step Delay (cycles)
95	None	00:05:00	4.40		0	0	0

Program Name	Program		
Cycles	40	Analysis Mode	Quantification

Target (°C)	Acquisition Mode	Hold (hh:mm:ss)	Ramp Rate (°C/s)	Acquisitions (per °C)	Sec Target (°C)	Step size (°C)	Step Delay (cycles)
95	None	00:00:30	4.40		0	0	0
60	Single	00:00:30	2.20		0	0	0

Program Name	Program		
Cycles	1	Analysis Mode	None

Target (°C)	Acquisition Mode	Hold (hh:mm:ss)	Ramp Rate (°C/s)	Acquisitions (per °C)	Sec Target (°C)	Step size (°C)	Step Delay (cycles)
37	None	00:00:10	2.20		0	0	0

Samples

Sample Count	96
Subset	All Samples

Pos	Name	ID	Repl. Of	Sample Notes
A1	K545			
A2	K5			
A3	Sample 3			
A4	Sample 4			
A5	Sample 5			
A6	Sample 6			
A7	Sample 7			
A8	Sample 8			
A9	Sample 9			
A10	Sample 10			
A11	Sample 11			
A12	Sample 12			
B1	K568			
B2	Sample 14			
B3	Sample 15			
B4	Sample 16			
B5	Sample 17			
B6	Sample 18			
B7	Sample 19			
B8	Sample 20			
B9	Sample 21			
B10	Sample 22			
B11	Sample 23			
B12	Sample 24			
C1	K612			
C2	K149			
C3	Sample 27			
C4	Sample 28			
C5	Sample 29			
C6	Sample 30			
C7	Sample 31			
C8	Sample 32			
C9	Sample 33			
C10	Sample 34			
C11	Sample 35			
C12	Sample 36			
D1	K172			
D2	K138			
D3	Sample 39			

Samples

Sample Count	96
Subset	All Samples

Pos	Name	ID	Repl. Of	Sample Notes
D4	Sample 40			
D5	Sample 41			
D6	Sample 42			
D7	Sample 43			
D8	Sample 44			
D9	Sample 45			
D10	Sample 46			
D11	Sample 47			
D12	Sample 48			
E1	K163			
E2	Sample 50			
E3	Sample 51			
E4	Sample 52			
E5	Sample 53			
E6	Sample 54			
E7	Sample 55			
E8	Sample 56			
E9	Sample 57			
E10	Sample 58			
E11	Sample 59			
E12	T-			
F1	K12			
F2	Sample 62			
F3	Sample 63			
F4	Sample 64			
F5	Sample 65			
F6	Sample 66			
F7	Sample 67			
F8	Sample 68			
F9	Sample 69			
F10	Sample 70			
F11	Sample 71			
F12	T-			
G1	K23			
G2	Sample 74			
G3	Sample 75			
G4	Sample 76			
G5	Sample 77			
G6	Sample 78			

Samples

Sample Count	96
Subset	All Samples

Pos	Name	ID	Repl. Of	Sample Notes
G7	Sample 79			
G8	Sample 80			
G9	Sample 81			
G10	Sample 82			
G11	Sample 83			
G12	T-			
H1	K52			
H2	Sample 86			
H3	Sample 87			
H4	Sample 88			
H5	Sample 89			
H6	Sample 90			
H7	Sample 91			
H8	Sample 92			
H9	Sample 93			
H10	Sample 94			
H11	Sample 95			
H12	T+			

Revision History

Revision	Date	User	Reason

Abs Quant/Fit Points for Bor GLPQ Kayes d 31-07-23 (Abs Quant/Fit Points)

Results

Inc	Pos	Name	Type	CP	Concentration	Standard	Status
☑	A1	K545	Unknown				
☑	A2	K5	Unknown				
☑	B1	K568	Unknown				
☑	B2	Sample 14	Unknown	35.66			
☑	C1	K612	Unknown	37.99			
☑	C2	K149	Unknown	33.67			
☑	D1	K172	Unknown	36.12			
☑	D2	K138	Unknown	34.35			
☑	E1	K163	Unknown	34.61			
☑	E12	T-	Unknown				
☑	F1	K12	Unknown	36.63			

Results

Inc	Pos	Name	Type	CP	Concentration	Standard	Status
☑	F12	T-	Unknown				
☑	G1	K23	Unknown				
☑	G12	T-	Unknown				
☑	H1	K52	Unknown	36.88			
☑	H12	T+	Unknown	23.43			

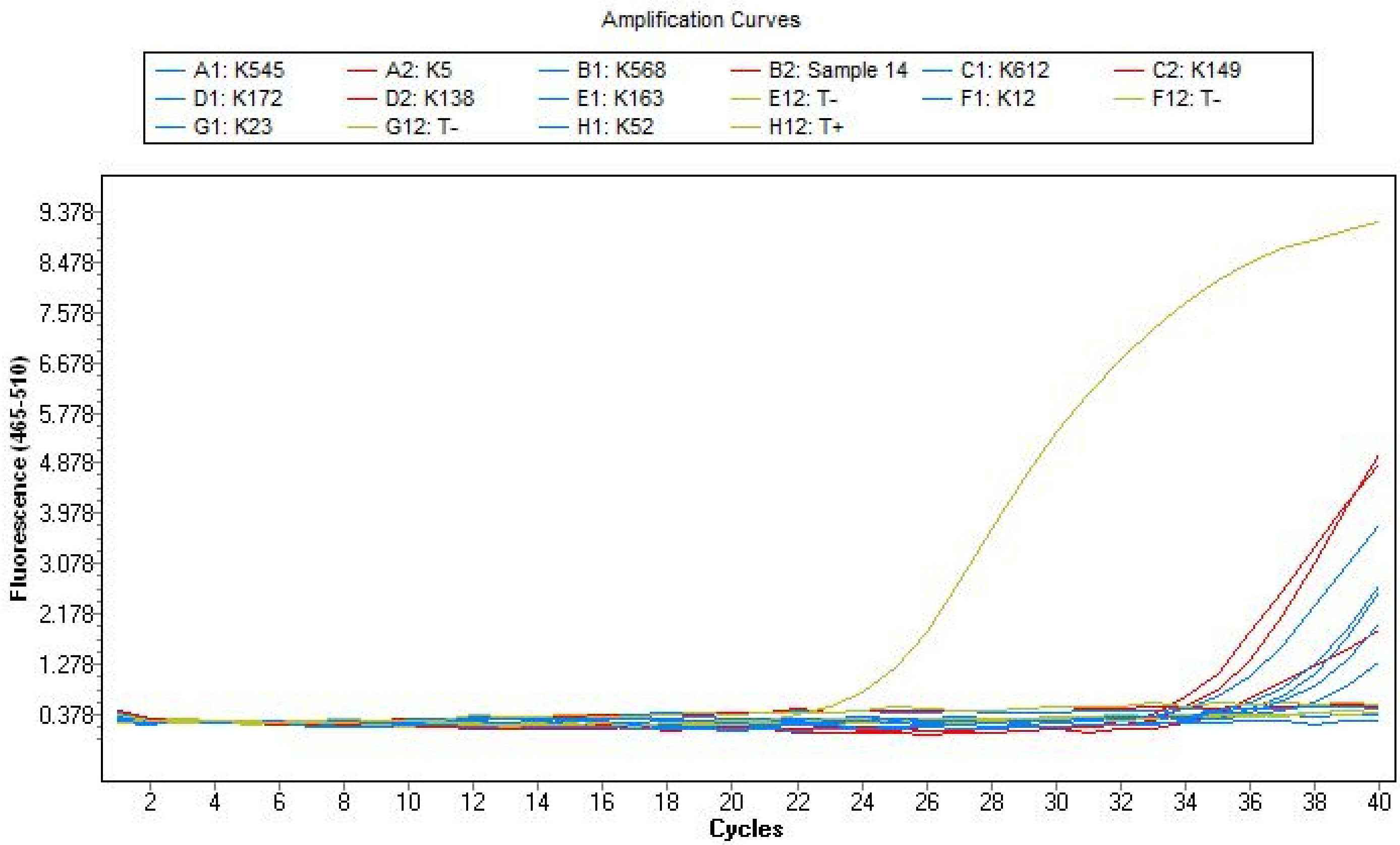

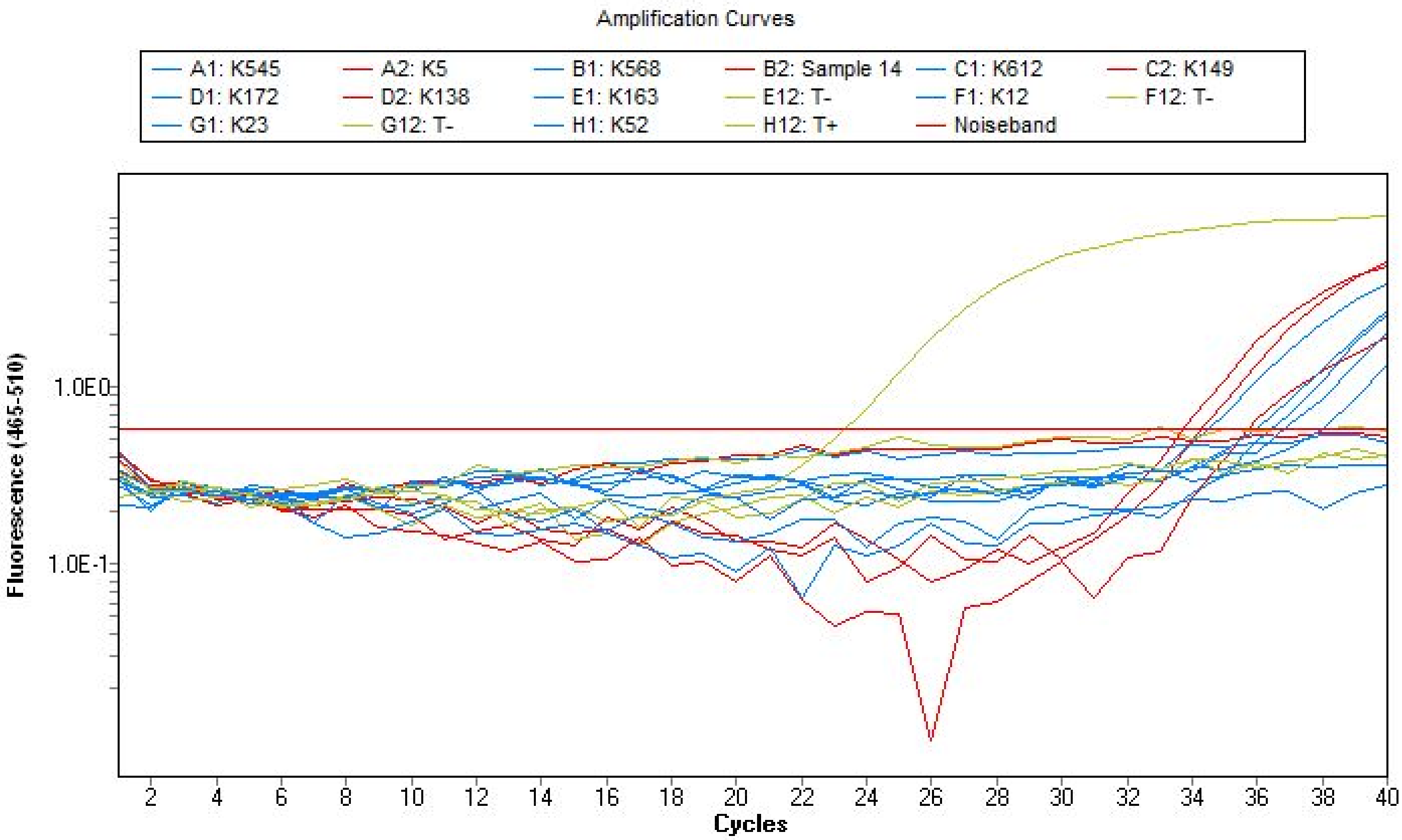
Amplification Curves
A1: K545
A2: K5
B1: K568
B2: Sample 14
C1: K612
C2: K149
D1: K172
D2: K138
E1: K163
E12: T-
F1: K12
F12: T-
G1: K23
G12: T-
H1: K52
H12: T+
Noiseband
Fluorescence (465-510)
1.0E0
1.0E-1
2 4 6 8 10 12 14 16 18 20 22 24 26 28 30 32 34 36 38 40
Cycles
