# Supplementary material for: Quantitative polymerase chain reaction from malaria rapid diagnostic tests to detect Borrelia crocidurae in Mali
Source: PLoS Negl Trop Dis. 2026 Aug 10;20(8):e0014033. doi: 10.1371/journal.pntd.0014033 (PMC13521451; doi:10.1371/journal.pntd.0014033)
Supplement: Supplementary file 2 [file pntd.0014033.s002.pdf]

Pascal DEMBELE Borrelia du 19-05-2023

Programs

Program Name	Program		
Cycles	1	Analysis Mode	None

Target (°C)	Acquisition Mode	Hold (hh:mm:ss)	Ramp Rate (°C/s)	Acquisitions (per °C)	Sec Target (°C)	Step size (°C)	Step Delay (cycles)
50	None	00:02:00	4.40		0	0	0

Program Name	Program		
Cycles	1	Analysis Mode	None

Target (°C)	Acquisition Mode	Hold (hh:mm:ss)	Ramp Rate (°C/s)	Acquisitions (per °C)	Sec Target (°C)	Step size (°C)	Step Delay (cycles)
95	None	00:05:00	4.40		0	0	0

Program Name	Program		
Cycles	40	Analysis Mode	Quantification

Target (°C)	Acquisition Mode	Hold (hh:mm:ss)	Ramp Rate (°C/s)	Acquisitions (per °C)	Sec Target (°C)	Step size (°C)	Step Delay (cycles)
95	None	00:00:30	4.40		0	0	0
60	Single	00:00:30	2.20		0	0	0

Program Name	Program		
Cycles	1	Analysis Mode	None

Target (°C)	Acquisition Mode	Hold (hh:mm:ss)	Ramp Rate (°C/s)	Acquisitions (per °C)	Sec Target (°C)	Step size (°C)	Step Delay (cycles)
37	None	00:00:10	2.20		0	0	0

Revision History

Revision	Date	User	Reason

Abs Quant/Fit Points for All Samples (Abs Quant/Fit Points)

Results

Inc	Pos	Name	Type	CP	Concentration	Standard	Status
☑	A1	KP1	Unknown				
☑	A2	KP9	Unknown				
☑	A3	KP17	Unknown				
☑	A4	KP25	Unknown				
☑	A5	KX2	Unknown				

Results

Inc	Pos	Name	Type	CP	Concentration	Standard	Status
☑	A6	K12	Unknown	32.92			
☑	A7	K49	Unknown				
☑	A8	K149	Unknown	35.95			
☑	A9	Sample 9	Unknown				
☑	A10	Sample 10	Unknown				
☑	A11	Sample 11	Unknown				
☑	A12	Sample 12	Unknown				
☑	B1	KP2	Unknown				
☑	B2	KP10	Unknown				
☑	B3	KP18	Unknown				
☑	B4	KP26	Unknown				
☑	B5	KX3	Unknown				
☑	B6	K28	Unknown				
☑	B7	K50	Unknown				
☑	B8	K155	Unknown				
☑	B9	Sample 21	Unknown				
☑	B10	Sample 22	Unknown				
☑	B11	Sample 23	Unknown				
☑	B12	Sample 24	Unknown				
☑	C1	KP3	Unknown				
☑	C2	KP11	Unknown				
☑	C3	KP19	Unknown				
☑	C4	K77	Unknown				
☑	C5	KX4	Unknown				
☑	C6	K32	Unknown				
☑	C7	K51	Unknown				
☑	C8	K158	Unknown	36.16			
☑	C9	Sample 33	Unknown				
☑	C10	Sample 34	Unknown				
☑	C11	Sample 35	Unknown				
☑	C12	Sample 36	Unknown				
☑	D1	KP4	Unknown				
☑	D2	KP12	Unknown				
☑	D3	KP20	Unknown				
☑	D4	K78	Unknown				
☑	D5	KX5	Unknown	35.54			
☑	D6	K57	Unknown				

Results

Inc	Pos	Name	Type	CP	Concentration	Standard	Status
☑	D7	K89	Unknown				
☑	D8	K160	Unknown				
☑	D9	Sample 45	Unknown				
☑	D10	Sample 46	Unknown				
☑	D11	Sample 47	Unknown				
☑	D12	Sample 48	Unknown				
☑	E1	KP5	Unknown				
☑	E2	KP13	Unknown				
☑	E3	KP21	Unknown				
☑	E4	K79	Unknown				
☑	E5	KX6	Unknown				
☑	E6	K67	Unknown				
☑	E7	K113	Unknown				
☑	E8	T-	Unknown				
☑	E9	Sample 57	Unknown				
☑	E10	Sample 58	Unknown				
☑	E11	Sample 59	Unknown				
☑	E12	Sample 60	Unknown				
☑	F1	KP6	Unknown				
☑	F2	KP14	Unknown				
☑	F3	KP22	Unknown				
☑	F4	K65	Unknown				
☑	F5	KX7	Unknown				
☑	F6	K72	Unknown	36.27			
☑	F7	K121	Unknown				
☑	F8	T-	Unknown				
☑	F9	Sample 69	Unknown				
☑	F10	Sample 70	Unknown				
☑	F11	Sample 71	Unknown				
☑	F12	Sample 72	Unknown				
☑	G1	KP7	Unknown				
☑	G2	KP15	Unknown				
☑	G3	KP23	Unknown				
☑	G4	K66	Unknown				
☑	G5	KX8	Unknown				
☑	G6	K73	Unknown				
☑	G7	K129	Unknown				

Results

Inc	Pos	Name	Type	CP	Concentration	Standard	Status
☑	G8	T-	Unknown				
☑	G9	Sample 81	Unknown				
☑	G10	Sample 82	Unknown				
☑	G11	Sample 83	Unknown				
☑	G12	Sample 84	Unknown				
☑	H1	KP8	Unknown				
☑	H2	KP16	Unknown				
☑	H3	KP24	Unknown				
☑	H4	KX1	Unknown				
☑	H5	K35	Unknown				
☑	H6	K39	Unknown				
☑	H7	K137	Unknown				
☑	H8	T+	Unknown	28.54			
☑	H9	Sample 93	Unknown				
☑	H10	Sample 94	Unknown				
☑	H11	Sample 95	Unknown				
☑	H12	Sample 96	Unknown				

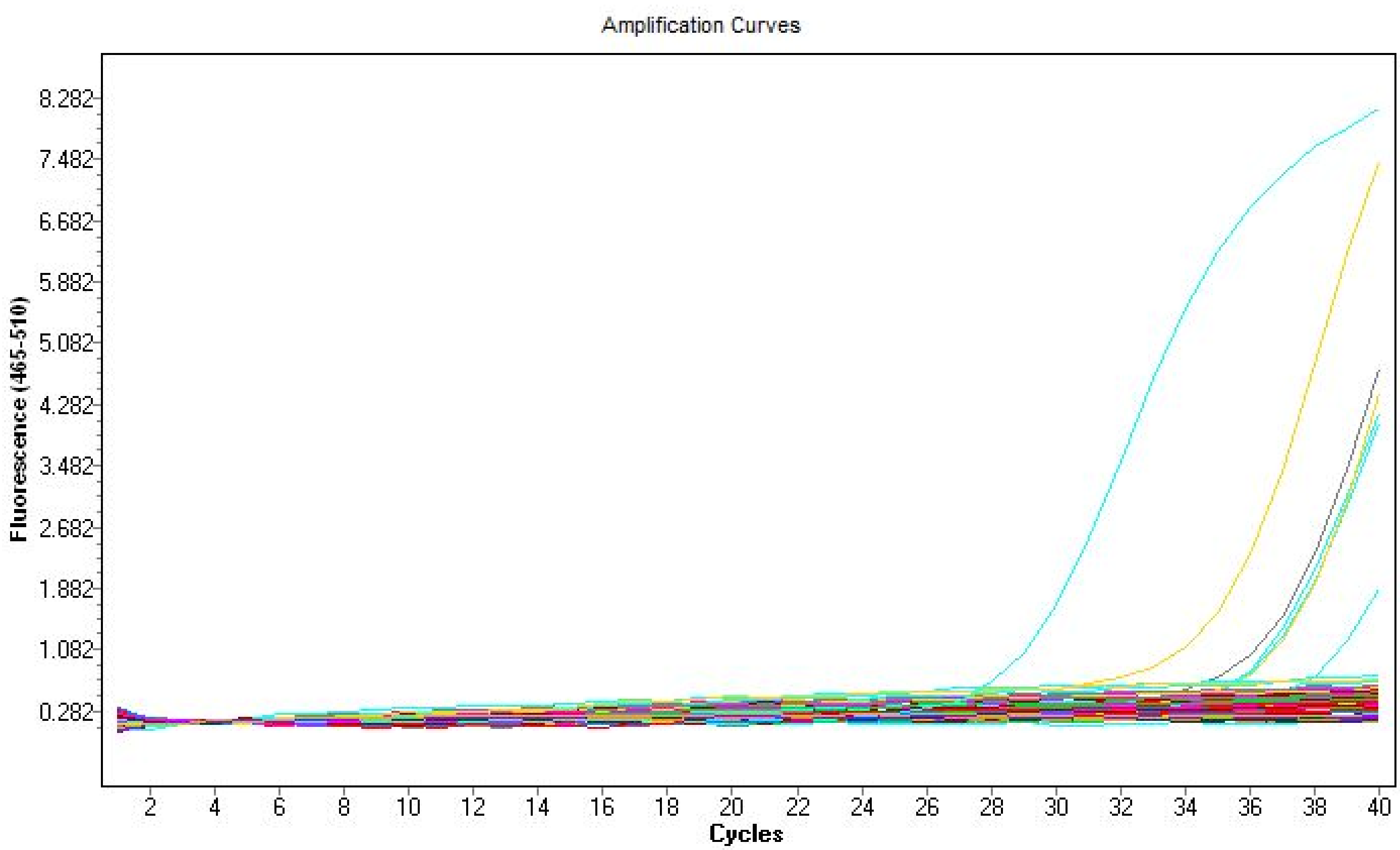

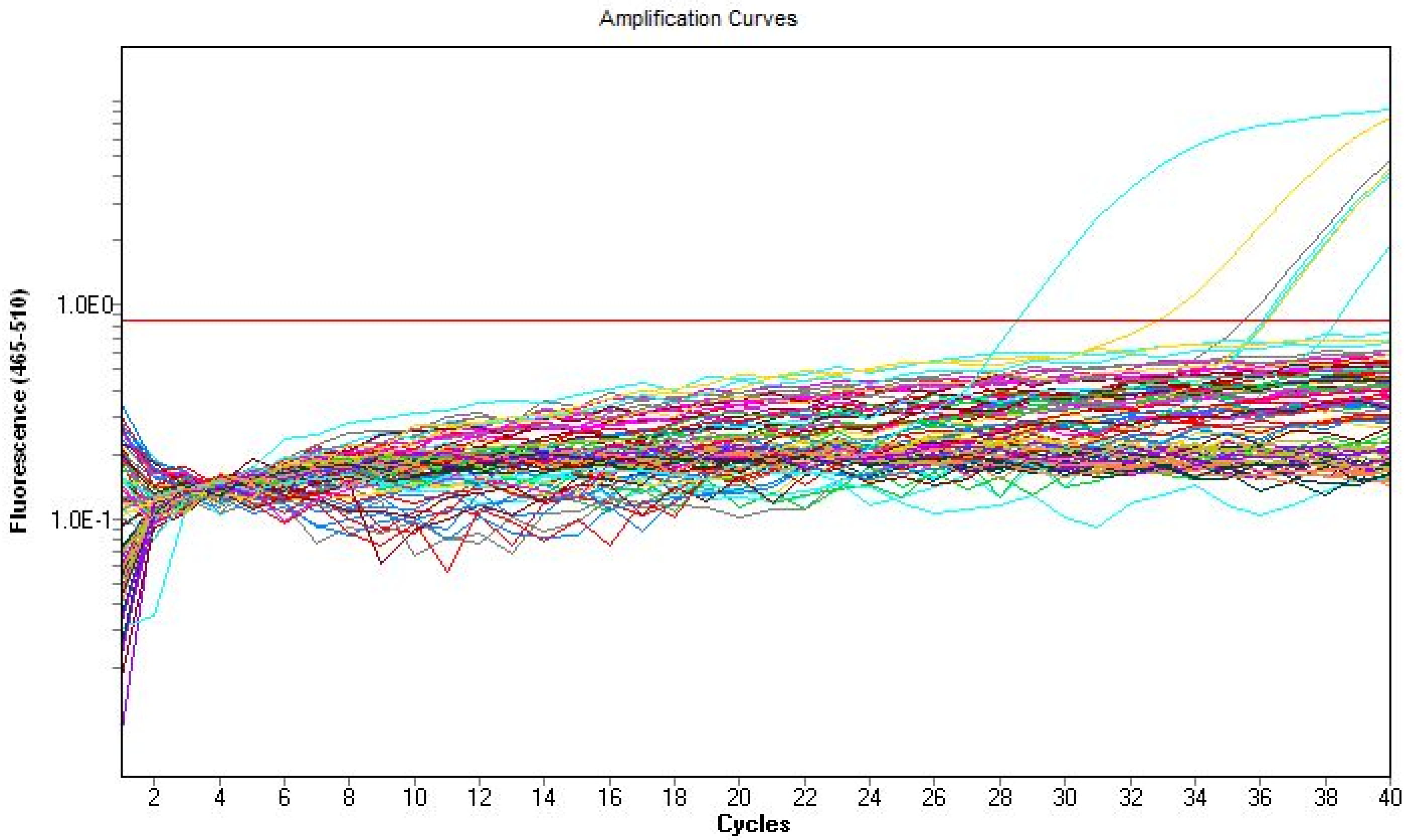

Abs Quant/Fit Points for Pascal Borrelia 190523 (Abs Quant/Fit Points)

Results

Inc	Pos	Name	Type	CP	Concentration	Standard	Status
☑	A1	KP1	Unknown				
☑	A2	KP9	Unknown				
☑	A3	KP17	Unknown				
☑	A4	KP25	Unknown				
☑	A5	KX2	Unknown				
☑	A6	K12	Unknown	32.49			
☑	A7	K49	Unknown				
☑	A8	K149	Unknown	35.84			
☑	B1	KP2	Unknown				
☑	B2	KP10	Unknown				
☑	B3	KP18	Unknown				
☑	B4	KP26	Unknown				
☑	B5	KX3	Unknown				
☑	B6	K28	Unknown				
☑	B7	K50	Unknown				
☑	B8	K155	Unknown				
☑	C1	KP3	Unknown				

Results

Inc	Pos	Name	Type	CP	Concentration	Standard	Status
☑	C2	KP11	Unknown				
☑	C3	KP19	Unknown				
☑	C4	K77	Unknown				
☑	C5	KX4	Unknown				
☑	C6	K32	Unknown				
☑	C7	K51	Unknown				
☑	C8	K158	Unknown	35.94			
☑	D1	KP4	Unknown				
☑	D2	KP12	Unknown				
☑	D3	KP20	Unknown				
☑	D4	K78	Unknown				
☑	D5	KX5	Unknown	35.25			
☑	D6	K57	Unknown				
☑	D7	K89	Unknown				
☑	D8	K160	Unknown				
☑	E1	KP5	Unknown				
☑	E2	KP13	Unknown				
☑	E3	KP21	Unknown				
☑	E4	K79	Unknown				
☑	E5	KX6	Unknown				
☑	E6	K67	Unknown				
☑	E7	K113	Unknown				
☑	E8	T-	Unknown				
☑	F1	KP6	Unknown				
☑	F2	KP14	Unknown				
☑	F3	KP22	Unknown				
☑	F4	K65	Unknown				
☑	F5	KX7	Unknown				
☑	F6	K72	Unknown	35.97			
☑	F7	K121	Unknown				
☑	F8	T-	Unknown				
☑	G1	KP7	Unknown				
☑	G2	KP15	Unknown				
☑	G3	KP23	Unknown				
☑	G4	K66	Unknown				
☑	G5	KX8	Unknown				
☑	G6	K73	Unknown				

Results

Inc	Pos	Name	Type	CP	Concentration	Standard	Status
☑	G7	K129	Unknown				
☑	G8	T-	Unknown				
☑	H1	KP8	Unknown				
☑	H2	KP16	Unknown				
☑	H3	KP24	Unknown				
☑	H4	KX1	Unknown				
☑	H5	K35	Unknown				
☑	H6	K39	Unknown				
☑	H7	K137	Unknown				
☑	H8	T+	Unknown	28.29			

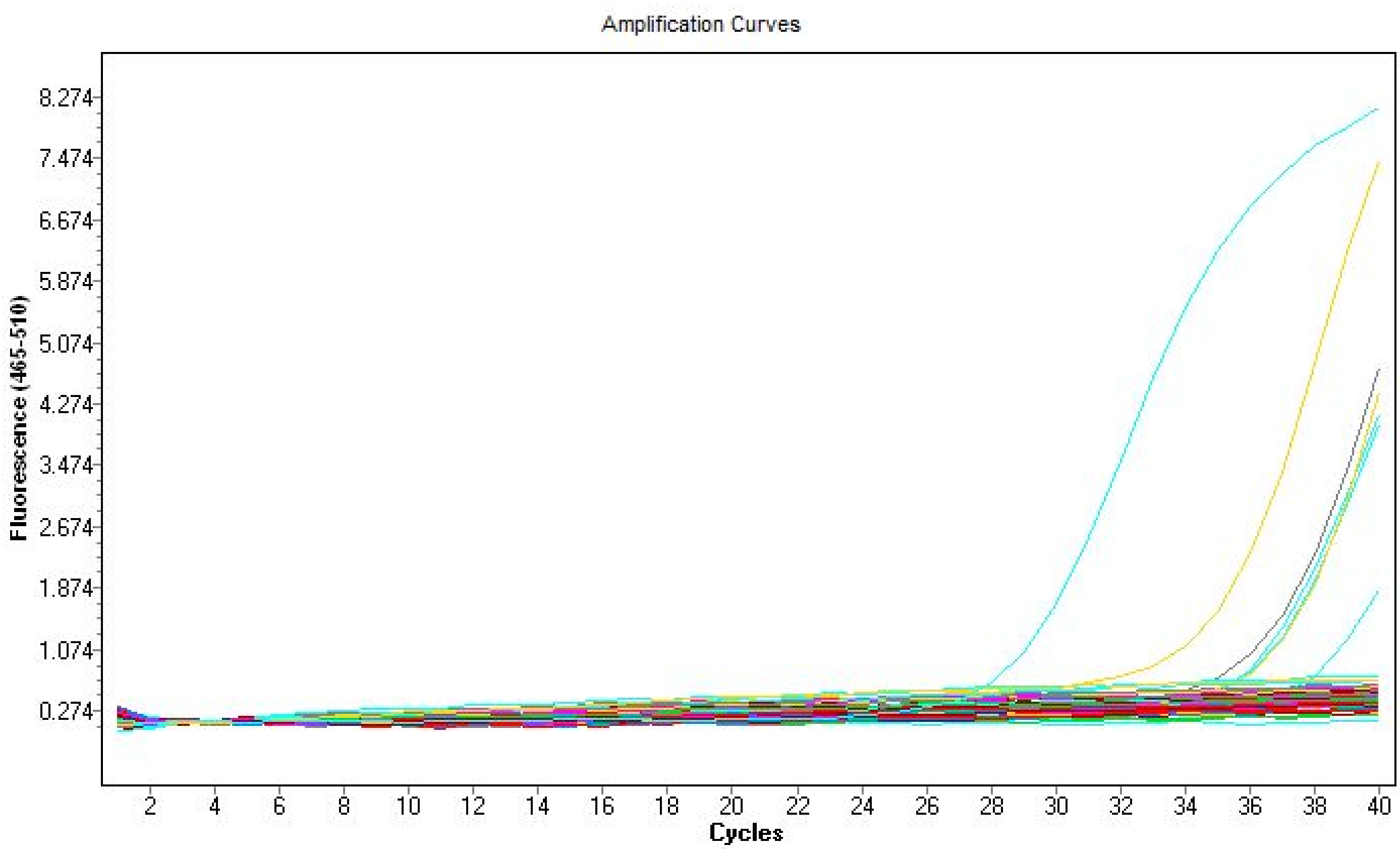

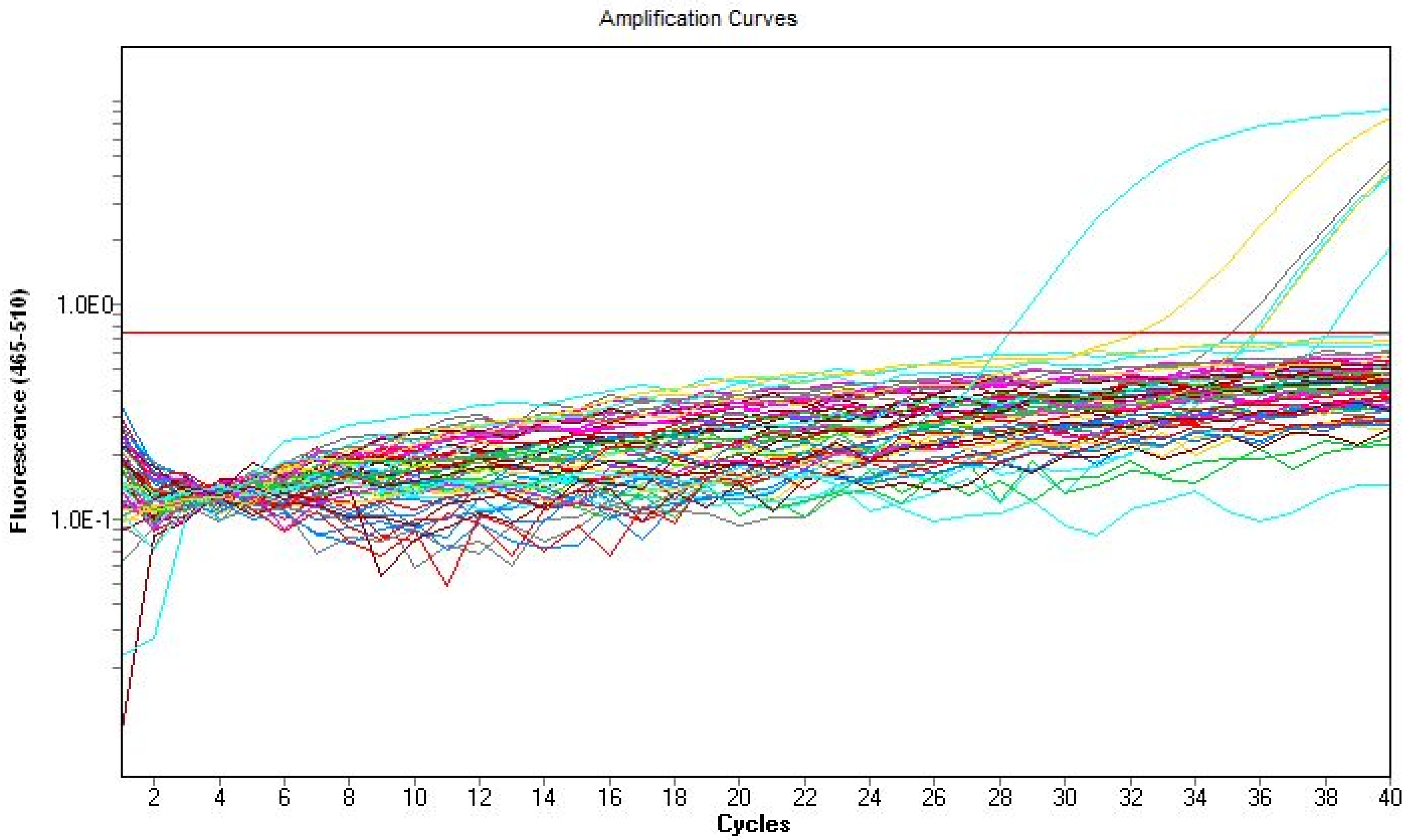
Amplification Curves
1.0E0
1.0E-1
Fluorescence (465-510)
2
4
6
8
10
12
14
16
18
20
22
24
26
28
30
32
34
36
38
40
Cycles
