# Supplementary material for: Quantitative polymerase chain reaction from malaria rapid diagnostic tests to detect Borrelia crocidurae in Mali
Source: PLoS Negl Trop Dis. 2026 Aug 10;20(8):e0014033. doi: 10.1371/journal.pntd.0014033 (PMC13521451; doi:10.1371/journal.pntd.0014033)
Supplement: Supplementary file 3 [file pntd.0014033.s004.pdf]

TO EDITOR OF PLOS NEGLECTED TROPICAL DISEASES

MANUSCRIPT TITLE: Quantitative polymerase chain reaction from malaria rapid diagnostic tests to detect *Borrelia crocidurae* in febrile patients in Mali

MANUSCRIPT ID: PNTD-D-26-00231

Name of Reviewer: Dr. Georges DIATTA

The manuscript PNTD-D-26-00231 entitled "Quantitative polymerase chain reaction from malaria rapid diagnostic tests to detect *Borrelia crocidurae* in febrile patients in Mali" is an interesting retrospective study performed by Dembélé *et a*l. that reported for the first time human cases of tick-borne relapsing fever (TBRF) in Mali using malaria rapid diagnostic tests (RDTs) collected in health centers from 9 regions for monitoring the occurrence of borreliosis cases caused by *Borrelia crocidurae* between June to December 2023. The malaria RDTs usually taken for detecting, for example *Plasmodium falciparum* –extracellular parasite- in febrile patients were been demonstrated as a reliable source of bacterial DNA for the investigation of acute undifferentiated febrile illness or extracellular pathogens from neglected arthropod-borne tropical diseases. This is why they are used in the study to screen for the pathogen bacteria, *B. crocidura*, in endemic areas. The methodological approach used in this study made possible to specify the proportion of *Borrelia* infection in this western part of Mali, which is a major first in epidemiological finding of human cases of borreliosis of major importance. Data prevalence of positive *B. crocidurae* DNA extracts from malaria TDRs obtained confirmed the wide known geographical distribution of borreliosis in western Mali. Globally, the manuscript is clean, however should be better structured and written in several points especially in the abstract, description of malaria RDTs collection, results title and the discussion.

1. Major comments:

According to the submission guidelines of Journal Plos Neglected Tropical Diseases the abstract (with 250-300 words) is conceptually divided into three sections: (i) background, (ii) methodology/Principal findings, (iii) Conclusion/Significance. Please adopt this structuring, and present an author summary before the introduction. The malaria RDTs usually taken for detecting, for example *Plasmodium falciparum* –extracellular parasite- in febrile patients were been demonstrated as a reliable source of bacterial DNA for the investigation of acute undifferentiated febrile illness or extracellular pathogens from neglected arthropod-borne tropical diseases. This is why they are used in the study to screen for the pathogen bacteria, *B. crocidura*, in Mali endemic areas. In the manuscript specify anywhere, if

necessary, when you must write "malaria RDTs for *Plasmodium falciparum*" which includes malaria-positive and malaria negative RDTs for *P. falciparum* or of other species of malaria such as *P. ovale, P. vivax, P. malariae*, either "malaria-positive RDTs for *P. falciparum* (*P.* f) or malaria-negative RDTs for *P. falciparum*" or another malaria species targeted in febrile patients. In the manuscript we do not know the malaria RDTs for which species you collected in health centers in Mali. I imagine you have collected malaria RDTs for *P. falciparum* which are more commonly used, if both malaria-positive and malaria-negative RDTs *P. f* were sampled in health centers, please provide as much descriptive information as possible that concerning. In materials and methods, please replaces the subtitle "study sites" with "study areas", so in this subtile where you describe the climatic zones, they should be illustrated with a map representing Figure 1 at line 83 at the end of the sentence. In addition, malaria RDTs were collected in health centers, not in sites, then write rather "malaria RDTs were collected from 41 health centers in 9 regions by indicating at line 84 malaria RDTs for which species targeted (malaria RDTs *P. f*) you sampled as suggested above with a detailed description of the number of health centers per region where the "malaria RDTs were collected and illustrated with Figure 2, which replace Figure 1, and indicate that you must perform a qPCR *P. falciparum* to confirm and/or infirm respective positivity or negativity of DNA extracts from malaria-positive and malaria-negative RDTs *P. f* as false positive and false negative results are sometimes observed in malaria RDTs *P. f* tested, and also search cases of *Borrelia* positive DNA extracts from malaria RDTs *P. f* associated with other pathogens such as *P. falciparum*, *Coxiella burnetii*, *Bartonella quintana* and *Rickettsia* /*Anaplasma* spp.

In the results, structure a subtitle such as, (i) "Collection of malaria RDTs for *P. falciparum* in the targeted health centers", and describe, with a Table presented, all informations related to health centers targeted per region, geographic coordinates from health centers localities of each region, number of malaria-postive RDTs *P. f* or malaria-negative RDTs *P. f* collected and tested, then results of qPCR *P. falciparum* performed to confirm and/or infirm respective positivity or negativity of DNA extracts from malaria-positive and malaria-negative RDTs *P. f*, (ii) "Molecular detection of DNA extracts from malaria RDTs for *P. falciparum*", and describe all results of DNA extracts from malaria RDTs *P. f* tested for *Borrelia* infection both by *ITS4* and specific Borcro *glpQ* qPCR, and of possible cases of co-infections between *B. crocidurae* / *P. falciparum*, *B. crocidurae* / *C. burnetii*, *B. crocidurae* / *B. quintana*, *B. crocidurae* / *Rickettsia* /*Anaplasma* spp. researched.

In the discussion, from line 176 to 182, please remove as this argument does not concern and has no connection with the epidemiological context of TBRF in the endemic areas studied.
In addition, from line 169 to 170 remove inappropriate references [21,39,40] used to explain the impact of climate change, which unfortunately do not correspond to research on climate impacting TBRF, but rather concern Lyme disease and tick-borne encephalitis transmitted by hard species ticks of the genus *Ixodes*. The only studies known providing data climate influencing TBRF epidemiology were performed by Trape *et al*., 1996b.

2. Comment of minor points:
a /. Indicate the corresponding author with an asterisk * and mention his email address below the list of authors.
b/. In the abstract, at line 27, replace "sites" with "health centers".
At line 29 put a comma after the digit in the number 1496 as follows 1,496, then add after the number 9 "DNA extracts from" malaria-negative RDTs…??…., but after RDTs, specify which species of malaria is targeted (malaria RDTs *P. f*?), "for an overall prevalence of 0.6% (9/1,496)".
At line 30 to 31, the prevalence rate of *B. crocidurae* in Kayes (6%, 9/150) is not correct. Please correct with the right ratio 9/180 (5%) and specify after malaria RDTs species of malaria targeted (malaria RDTs *P. f*?).
At line 34, remove "Malaria" and add "malaria" in front of "RDTs *P. f*?" then specify species of malaria targeted (malaria RDTs *P. f*?).
c/. In the introduction, at line 37, the sentence is poorly worded, since *Ornithodoros* soft ticks are vectors, but not the cause of febrile infections due to *B. croicidurae*. Then remove the parentheses in TBRF at lines 37, 43 and 45. Rewrite the sentence at lines 37-38 as follows: "Transmitted recurrent fever like TBRF by soft ticks of the genus *Ornithodoros* are neglected febrile infections due to *Borrelia* spp."
At line 44, the reference [9] cited is related to louse-borne disease due to *Borrelia recurrentis*, please remove.
At line 45, *B. crocidurae* is not a vector, please reword the sentence.
At line 39, remove "caused by" and replace with "hosting", then delete "the genus" and add "species" after *Borrelia*.
Borrelia duttonii and *B. recurrentis* are not hosted by wild rodents, please remove.
At line 46, it is not true by saying "extreme precarious conditions" for rural populations living in endemic TBRF areas, but rather to rural populations living in traditional dwellings made of mud and sleeping on the floor. The references cited [10,11] not appropriate for TBRF in West Africa, please replace with Trape *et a*l., 1991, 1996, 2013.

At line 61, replace “proliferation” with “spread”, and line 62 replace also “responsible for” with “vector of”.
At line 67, blood smears are a tool (technique) for detecting malaria parasites, please so reword.
From line 71 to 72, add “Malaria” in front of rapid diagnostic tests (RDTs) in the beginning of the sentence and specify malaria species targeted (malaria RDTs for *P. falciparum* or use *P. f*?).
Not true to say “due to their easy use” in line 72, remove and write the right reason.
At line 74, it is likely that reference [28] is missing here, please verify. Then replace “DNA extracted from RDTs” with “DNA extracts from RDTs for *P. falciparum*” to…
At line 75, replace “fever in RDTs-negative” with “acute undifferentiated febrile illness" in patients in Mali.
d/. In materials and methods, replace “RDTs cassettes” with “malaria RDTs for *P. falciparum?*”, then replace “sites” with “health centers”. You say that malaria RDTs *P. f*? were collected from June to December 2021 at line 86, while the approval for the use of malaria RDTs (*P. f*?) has been granted in February 2023, please correct with the right date.
The description of climatic zones must be illustrated with a map showing climatic zones in Mali (title Figure 1 to cite at line 84, end of the sentence), so the Figure 1 becomes Figure 2 to cite at line 87, end of the sentence, a showing the localities where malaria RDTs for *P. falciparum*? were collected in health centers and results of molecular analysis, in Mali (title Figure 2 at line 142). Please mention “Legend” above Figure 2
Title Figure 1: Map of Mali showing climatic zones (at line 84)
Title Figure 2: Map of Mali showing the localities where malaria RDTs for *P. falciparum*? were collected in health centers and results of molecular analysis (at line 142), no malaria RDTS *P. f* collection data on the map, please remove all.
From line 78 to 87, lack of double spacing between lines in this paragraph.
At line 89, add after from “malaria RDTs *P. falciparum*” performed was ….
At line 110, remove “M” after Rahal, and line 120 the Figure 2 becomes Figure 3.
e/. In ethical considerations, at line 125 replace “RDTs” with “malaria RDTs for *P. falciparum*?”, and write correctly reference of approval granted under this number **064** /MSDS-SG/PNLP at line 129.
f/. In the results, please delete all numbers mentioned in the Figure 2 in each region as these data must be reported in a Table, then indicate legend of screening results. At line 135 Figure 1 becomes Figure 2.

Describe the results of title "Collection of malaria RDTs for *P. falciparum* in the targeted health centers", then those of that "Molecular detection of DNA extracts from malaria RDTs for *P. falciparum?*".
At line 140, Figure 2 becomes Figure 3. In this Figure 3 replace "Negative RDTs cassettes" with "Malaria-negative RDTs" for *P. falciparum*, then replace "Host tick" *O. sonrai* in Senegal Mali and Mauritania with "Vector tick" *O. sonrai* in Senegal Mali and Mauritania.
At line 150, the Figure 2 becomes Figure 3, then add "malaria-negative RDTs" *P. f*? after from.
Title Figure 3: Phylogenetic tree (maximum likelihood method, bootsrap 1,000) of partial sequence data for the flagellin gene (FlaB) of *B. crocidurae* identified from malaria-negative for *P.* falciparum (at line 150)
g/. In the discussion, at line 157 please provide corresponding reference related to this sentence.
At line 158, add "tick" after vector, and line 159, add "these" after "17.3% of".
At line 163 to 164, reword the sentence as follows: Our *B. crocidurae* prevalence found in Kayes (5%, 9/180) close to that of Mediannikov et. al (provide reference) conducted …….
At line 167, after "affected", remove the colon, and add "with 13.5% of *Borrelia* infection" (the report 33/173 mentioned is not correct, please provide the right one).
At line 169 to 170, provide appropriate reference.
At line 171, add "Senegal" after "In", then from line 171 to 175, provide appropriate reference related, end of the sentence.
At line 182, remove "In this context", and begin the sentence with "A similar study performed by Ndiaye et al.…., then remove "EHI" after Ndiaye at line 183. Add "DNA extracts from malaria RDTs *P. falciparum*? collected in Senegal" after prevalence of *B. crocidurae* in", line 183.
At line 185, after "detected in our", add "DNA extracts from malaria-negative RDTs *P. f*? samples was previously been identified"
At line 187, after "*the Borrelia*" add "detected" in our study
At line 195, after "the disease" add "in endemic rural areas from Mali and West African countries where molecular biology techniques are lacking" (to complete the sentence).
h/. In the references, at line 233 remove reference [9] concerning louse-borne disease.
At line 245 and line 247, reference duplicate, remove one of them [14].
At line 266, 320, 323, remove these inappropriate references [21,39,40].
At line 299 and line 302, reference duplicate, remove one of them [32].
