# Supplementary material for: Quantitative polymerase chain reaction from malaria rapid diagnostic tests to detect Borrelia crocidurae in Mali
Source: PLoS Negl Trop Dis. 2026 Aug 10;20(8):e0014033. doi: 10.1371/journal.pntd.0014033 (PMC13521451; doi:10.1371/journal.pntd.0014033)
Supplement: Supplementary file 4 [file pntd.0014033.s005.docx]

**Reviewer#1:**  **General Comments**

Dembele and coworkers investigated possible human infections with the tick-borne relapsing fever spirochete *Borrelia crocidurae* in the various administrative districts throughout Mali. Samples examined were collected previously on rapid diagnostic test strips used to detect Plasmodium antigens for the diagnosis of malaria. Blood was extracted and DNA was purified and tested with multiple qPCR and standard PCR assays. The partial flaB gene was amplified and sequenced from nine samples, all from the Kayes district of western Mali. The sequences produced were compared to sequences in GenBank and identified the organism detected as *Borrelia crocidurae*. Overall, the work is certainly worthy of investigation, the results are presented well with a few caveats listed below, and the conclusions are justified. My specific comments address items as they appear chronologically in the manuscript, so they are a mixture of minor to more significant issues.

**Authors’ response**: We would like to thank the reviewer for taking the time to review our article. We carefully took all comments and suggestions into account, as they help improve the quality of the manuscript.

**Specific Comments**

**1. Line 32** (abstract), “relapsed” should be relapsing.

**Authors’ response**: Thank you, as requested, we have replaced “relapsed” by “relapsing” in line 37.

**2. Line 37**, “Transmitted recurrent fevers (TBRF)” is not correct. TBRF stands for tick-borne relapsing fever, not transmitted recurrent fevers. The authors got it right almost everywhere else, but not here. Also, throughout the authors go back and forth with the full name and the abbreviation. Once tick-borne relapsing fever is abbreviated as TBRF, they should stick with this abbreviation.

**Authors’ response:** Thank you for your excellent observation. We have replaced “Transmitted recurrent fevers (TBRF)” by “Tick-borne relapsing fever” in line 60

**3. Line 37-38**, the authors state that TBRF are “febrile infections caused by soft ticks of the genus Ornithodoros sp.”. Tick do not cause the febrile infections, spirochetes do. Ticks transmit the spirochetes that cause the infection. This sentence needs to be fixed.
**Authors’ response:** The sentence has been modified as follows:

In line 60-61: “Tick-borne relapsing fever (TBRF) is a neglected febrile infection caused by spirochetes of the genus *Borrelia* and transmitted to humans by soft ticks of the genus *Ornithodoros*.”

**4. Line 45**, the authors state “*B. crocidurae* is recognized as the only vector responsible for (TBRF) disease”. B. crocidurae is not the vector but the causative agent, a spirochete. The authors need to fix this sentence.

**Authors’ response:** We agree with this remark. We have revised the sentence as follows:

In line 68-69: “In West Africa, B. crocidurae is recognized as the only causative agent of TBRF….”

**5. Line 61-62**, change “Ornithodoros sonrai responsible for *B. crocidurae*” to Ornithodoros sonai responsible for transmitting *B. crocidurae*”.

**Authors’ response:** We checked that *Ornithodoros sonrai* (Sautet & Witkowski, 1943) is the correct name, corresponding to ncbitaxon: 352064.

**6. Line 74-75**, the authors state “we used DNA extracted from RTDs to determine the prevalence of TBRF as a cause of fever in RDT-negative patients in Mali.” This is part of the presentation that I found a bit confusing. Were only malaria-negative samples tested, i.e. were (all malaria positive samples excluded? See below.

**Authors’ response:** Thank you for your excellent observation, we used DNA extracts from both positive and negative malaria RDTs please don’t only negative. The sentence has been modified as follows:

In line 102- 105: “DNA extracted from rapid diagnostic tests (RDTs), whether positive or negative for *P. falciparum*, was used to determine the prevalence of TBRF among patients with acute febrile illness in Mali”.

**7. Lines 84-86**, the authors state that they collected RDT cassettes from 41 sites in 9 administrative units. Because most of the units are large and can vary in their ecology, to me it would be worthwhile to list in a supplementary file what the 41 sites were. Part of the rationale for this is based on their reference 17, which found spirochete infected ticks and small mammals in the Bandiagara region of the Mopti District. Were sites included in this region that included TBRF endemic villages? In the Kayes District, were all (9) positive samples from the same district? If not too difficult, a table giving the sites would be of interest.
**Authors’ response**: Thank you very much for these different questions. We reassure you that the part concerning the collect of RDTs at the 41 health centres in 9 regions is summarized in a table 1. Unfortunately, we did not collected RDTs from Bandiagara district because of the growing insecurity in this part of central Mali. We only found the sample of tree sites of the Mopti distict (Soufouroulaye, Fatoma et Sevaré II) and Mopti is situated 73 km from Bandiagara.

Yes, all (9) positive samples from the same district Diema, site Diancounté camara in Kayes region. (Line 114-126)

**8. Lines 133-134**, the authors state “A total of 1496 malaria RDT samples (260 positive and 1236 negative from febrile patients were tested for Borrelia spp by real-time PCR.” Here then the authors apparently tested 260 positive malaria samples for *Borrelia* infection. Thus, this begs the question: were any dual infections found, i.e. patients with both plasmodium and borrelia infections?

A**uthors’ response:** We thank the reviewer for this comment. All 260 malaria-positive samples were tested for *Borrelia* infection. However, all samples were negative for *Borrelia*. Therefore, no cases of co-infection between *Plasmodium* and *Borrelia* were observed. We have added the following sentence to clarify this point:

Line 243: No cases of co-infections between B. crocidurae / P. falciparum.

**9.** In the results section, I think the authors should state how much flab sequence they determined and used in their alignments with homologous sequences they obtained from GenBank. The authors provide the nine partial flab sequences in a supplementary file, but the reader has to count the bases to determine the amount of sequence used. I did this, and the sequences ranged from 322 to 346 bases, with what appears to be good overlap among them. I think the authors should describe these sequences a bit more in the primary text of the article.
**Authors’ response:** As requested, we have included this information in the body of the article as follows:

In line 245-249: “Sequencing of the central region of the *flaB* gene yielded nine partial sequences ranging in length from 322 to 346 bp. Comparison of the central region of all partial *flaB* gene sequences of *Borrelia* obtained in this study with sequences in GenBank revealed 99–100% coverage and 100% identity with *B. crocidurae* sequences (JX119098, GU357619, and JX29291), detected, respectively, in blood in Senegal, in *Ornithodoros sonrai* in Mauritania, and in Mali”.

**10.** On a related issue, most journals require novel sequences to be deposited in GenBank with accession numbers for them included in the manuscript. This appears not to have been done. The editors might be best qualified to make this call.

**Authors’ response:** As requested, we have deposited our sequences in the GenBank database. This was indicated in the manuscript as follows:

Lines 252-253: “The partial *flaB* gene sequences of *B. crocidurae* detected in this study were deposited in GenBank under the following accession numbers: PZ232043–PZ232051.”

**11. Lines 138-140**, the authors state “The phylogenetic tree shows that our sequences are close to those already found in Mali, Mauritania and Senegal”. I think the authors should state what the percent identity values are. Also, it might be reasonable to state exactly how much of the partial flaB sequences were compared.

**Authors’ response:** Thank you. As requested, we have added the percent identity values and specified which regions of the *flaB* partial sequences were compared. Please see the sentence above for the answer to the question 9.

**12. Lines 142-143**, the authors give in the figure legend “Map of Mali showing the number of malaria RDT cassettes tested in the denominator and the number positive in the numerator.” For clarity, I suggest changing this to “Map of Mali showing the ten administrative districts and the number of malaria RDT cassettes tested in the denominator and the number of *Borrelia* positive in the numerator.”

**Authors’ response:** Thank you for your suggestion. As recommended, we have revised the legend of Figure 2 as follows:

In line 255-256: “Map of Mali showing the localities where malaria RDTs for *P. falciparum* were collected in health centers and results of molecular analysis”.

On the contrary the Reviewer2 suggested that we remove the numbers on this map along with the title suggestion above. Thank you for your understanding.

**13. Line 152**, the authors state “Sequences of the *B. crocidurae* FlaB gene found in O. sonrai ticks”. FlaB should be flaB and the authors should reference the work finding *B. crocidurae* in these ticks.

**Authors’ response:** As suggested *“FlaB”* was replaced by *“flaB”.* However, regarding the addition of a reference, these sequences were obtained from our RDT samples. For clarity and accuracy, the sentence has been modified as follows:

In line 262-265 “A phylogenetic tree constructed using the maximum likelihood method with 1,000 bootstrap replicates, based on partial sequences of the flagellin (flaB) gene from B. crocidurae generated from RTD samples in this study. Sequences from several Borrelia species obtained from GenBank were included in the phylogenetic tree for comparison.”

**14. Lines 157-159**, the authors state for reference 17 that “a study conducted in 20 villages in southern Mali reported the presence of the vector O. sonrai in small mammals and ticks collected from rodent burrows”. This is awkward and should be changed to “the presence of the vector O. sonrai in small mammal burrows”. Delete “and ticks collected from rodent”. You don’t find O. sonrai in small mammals.

**Authors’ response:** The reviewer is correct, and we have revised the sentence as follows**:**

In line 268- 270: “Between December 2007 and October 2011, a study conducted in 20 villages in southern Mali reported the presence of the tick vector *O. sonrai* in rodent burrows, with 17.3% of the collected ticks infected with *B. crocidurae*.”

**15. Lines 175-176**, the authors state “The increase in the number of cases demonstrates that TBRF is a real public health problem in Senegal [reviewer, I agree] and other neighboring countries such as Mali.” This may be premature to make this claim for Mali given the information presented, nine human infections out of nearly 1500 samples and all restricted to the Kayes district in western Mali. Clearly more work needs to be done.

**Authors’ response:** We thank the reviewer for this comment and fully agree. The sentence has been revised as follows:

In line 286-290: “The increase in the number of cases indicates that tick-borne relapsing fever (TBRF) is a serious public health problem in Senegal. However, in Mali, a neighboring country, further studies are needed to determine the actual impact of TBRF on public health, as the present study detected only nine cases of *B. crocidurae* DNA, all of which were found exclusively in the Kayes region in western Mali.”

**16. Lines 187-188**, the authors state that the results of their study “were similar to those previously found in Senegal [17, 42].” Their reference 17 includes work done in Mali, so Mali should be included at the end of this sentence.

**Authors’ response:** The reviewer is correct. Mali has been included at the end of the sentence as follows:

In line 294-295: “Phylogenetic analysis of the *flaB* gene showed that the *Borrelia* in our study were similar to those previously found in Senegal and Mali.”

17. Line 325, the reference is incomplete. The journal, volume and pages need to be added.

**Authors’ response:** This reference was removed from the article because a reviewer 2 suggested it was inappropriate.

**18.** The paper is relatively short, so I think it would be helpful to expand a bit on the methods of how the samples were obtained and what the malaria RDT cassettes provided. I realize the authors cite a paper but one that is not the easiest to access. Did the authors go to major health clinics in all the administrative districts and obtain stored RDT cassettes, sampling 180 at random in each district? What was the time between when the malaria tests were performed and when the blood was eluded and DNA extracted? Were the cassettes held at room temperatures, which can be quite warm in many of the regions sampled. I think a more detailed description of the sampling would be useful and informative.

**Authors’ response:** We appreciate your excellent comments and feedback. We have made the necessary corrections to ensure that readers can clearly understand the methodology, as you suggested (line 118-125):

“The authors were unable to visit all the health centers where *P. falciparum* TDRs were collected due to the insecurity the country has experienced since 2012. PD is a national malaria control program officer responsible for epidemiological surveillance of malaria, and thanks to good collaboration, he obtained the TDRs from certain health centers located in areas that are very difficult to access

The time elapsed between the test and DNA extraction can be estimated at one year. It must be acknowledged that storage conditions are not always optimal after the RDTs have been used. Dust, heat, and humidity could reduce the quantity of the samples.

All *P. falciparum* malaria screening RDTs were stored at room temperature from the time of collection until DNA extraction at the laboratory of the IHU Méditerranée Infection in Marseille, France.”

**19.** Finally, possibly a table would be quite helpful that breaks down the samples as to how many malaria positive samples, malaria negative samples, borrelia positive samples and borrelia negative samples among them etc were done. Maybe not critical but I think the authors should consider such a table in their results section.

**Authors’ response:** Thank you very much for the suggestion. We have included a table titled “Detail of the total number and prevalence of positive and negative Malaria Rapid Diagnostic Tests in each health center,” as well as a paragraph on the “Collection of malaria RDTs in the targeted health centers.”(line 229- 234).
