# Supplementary material for: Quantitative polymerase chain reaction from malaria rapid diagnostic tests to detect Borrelia crocidurae in Mali
Source: PLoS Negl Trop Dis. 2026 Aug 10;20(8):e0014033. doi: 10.1371/journal.pntd.0014033 (PMC13521451; doi:10.1371/journal.pntd.0014033)
Supplement: Peer review history

# Peer review: Decision Letter 0

**DOI:** 10.1371/journal.pntd.0014033.r001

**Authors:** Joseph M. Vinetz

9 Mar 2026

Response to Reviewers Revised Manuscript with Track Changes Manuscript

Shaden Kamhawi

co-Editor-in-Chief

PLOS Neglected Tropical Diseases

orcid.org/0000-0003-4304-636XX

Paul Brindley

co-Editor-in-Chief

PLOS Neglected Tropical Diseases

orcid.org/0000-0003-1765-0002

**Journal Requirements:**

1) Please provide an Author Summary. This should appear in your manuscript between the Abstract (if applicable) and the Introduction, and should be 150-200 words long. The aim should be to make your findings accessible to a wide audience that includes both scientists and non-scientists. Sample summaries can be found on our website under Submission Guidelines:

https://journals.plos.org/plosntds/s/submission-guidelines#loc-parts-of-a-submission

2) Please upload all main figures as separate Figure files in .tif or .eps format. For more information about how to convert and format your figure files please see our guidelines:

https://journals.plos.org/plosntds/s/figures

3) We have noticed that you have uploaded Supporting Information files, but you have not included a list of legends. Please add a full list of legends for your Supporting Information files after the references list.

4) Some material included in your submission may be copyrighted. According to PLOSu2019s copyright policy, authors who use figures or other material (e.g., graphics, clipart, maps) from another author or copyright holder must demonstrate or obtain permission to publish this material under the Creative Commons Attribution 4.0 International (CC BY 4.0) License used by PLOS journals. Please closely review the details of PLOSu2019s copyright requirements here: PLOS Licenses and Copyright. If you need to request permissions from a copyright holder, you may use PLOS's Copyright Content Permission form.

Please respond directly to this email and provide any known details concerning your material's license terms and permissions required for reuse, even if you have not yet obtained copyright permissions or are unsure of your material's copyright compatibility. Once you have responded and addressed all other outstanding technical requirements, you may resubmit your manuscript within Editorial Manager.

Potential Copyright Issues:

- Please confirm (a) that you are the photographer of Figure 2, or (b) provide written permission from the photographer to publish the photo(s) under our CC BY 4.0 license.

- Figure 1: Please (a) provide a direct link to the base layer of the map (i.e., the country or region border shape) and ensure this is also included in the figure legend; and (b) provide a link to the terms of use / license information for the base layer image or shapefile. We cannot publish proprietary or copyrighted maps (e.g. Google Maps, Mapquest) and the terms of use for your map base layer must be compatible with our CC BY 4.0 license.

Note: if you created the map in a software program like R or ArcGIS, please locate and indicate the source of the basemap shapefile onto which data has been plotted.

If your map was obtained from a copyrighted source please amend the figure so that the base map used is from an openly available source. Alternatively, please provide explicit written permission from the copyright holder granting you the right to publish the material under our CC BY 4.0 license.

If you are unsure whether you can use a map or not, please do reach out and we will be able to help you. The following websites are good examples of where you can source open access or public domain maps:

* U.S. Geological Survey (USGS) - All maps are in the public domain. (http://www.usgs.gov)

* PlaniGlobe - All maps are published under a Creative Commons license so please cite u201cPlaniGlobe, http://www.planiglobe.com, CC BY 2.0u201d in the image credit after the caption. (http://www.planiglobe.com/?lang=enl)

* Natural Earth - All maps are public domain. (http://www.naturalearthdata.com/about/terms-of-use/).

- The following file contains screenshots: "supporting information file". We are not permitted to publish these under our CC-BY 4.0 license, websites are usually intellectual property and are copyrighted.This includes peripheral graphics of the web browser such as icons and button. We ask that you please remove or replace it.

5) Please amend your detailed Financial Disclosure statement. This is published with the article. It must therefore be completed in full sentences and contain the exact wording you wish to be published.

- State the initials, alongside each funding source, of each author to receive each grant. For example: "This work was supported by the National Institutes of Health (####### to AM; ###### to CJ) and the National Science Foundation (###### to AM)."

- State what role the funders took in the study. If the funders had no role in your study, please state: "The funders had no role in study design, data collection and analysis, decision to publish, or preparation of the manuscript.".

If you did not receive any funding for this study, please simply state: u201cThe authors received no specific funding for this work.u201d

**Reviewers' comments:**

**Key Review Criteria Required for Acceptance?**

As you describe the new analyses required for acceptance, please consider the following:

**Methods**

-Are the objectives of the study clearly articulated with a clear testable hypothesis stated?

-Is the study design appropriate to address the stated objectives?

-Is the population clearly described and appropriate for the hypothesis being tested?

-Is the sample size sufficient to ensure adequate power to address the hypothesis being tested?

-Were correct statistical analysis used to support conclusions?

-Are there concerns about ethical or regulatory requirements being met?

Reviewer #1: (No Response)

Reviewer #2: (No Response)

**********

**Results**

-Does the analysis presented match the analysis plan?

-Are the results clearly and completely presented?

-Are the figures (Tables, Images) of sufficient quality for clarity?

Reviewer #1: (No Response)

Reviewer #2: (No Response)

**********

**Conclusions**

-Are the conclusions supported by the data presented?

-Are the limitations of analysis clearly described?

-Do the authors discuss how these data can be helpful to advance our understanding of the topic under study?

-Is public health relevance addressed?

Reviewer #1: (No Response)

Reviewer #2: (No Response)

**********

**Editorial and Data Presentation Modifications?**

Use this section for editorial suggestions as well as relatively minor modifications of existing data that would enhance clarity. If the only modifications needed are minor and/or editorial, you may wish to recommend “Minor Revision” or “Accept”.

Reviewer #1: (No Response)

Reviewer #2: (No Response)

**********

**Summary and General Comments**

Use this section to provide overall comments, discuss strengths/weaknesses of the study, novelty, significance, general execution and scholarship. You may also include additional comments for the author, including concerns about dual publication, research ethics, or publication ethics. If requesting major revision, please articulate the new experiments that are needed.

Reviewer #1: General Comments

Dembele and coworkers investigated possible human infections with the tick-borne relapsing fever spirochete Borrelia crocidurae in the various administrative districts throughout Mali. Samples examined were collected previously on rapid diagnostic test strips used to detect Plasmodium antigens for the diagnosis of malaria. Blood was extracted and DNA was purified and tested with multiple qPCR and standard PCR assays. The partial flaB gene was amplified and sequenced from nine samples, all from the Kayes district of western Mali. The sequences produced were compared to sequences in GenBank and identified the organism detected as Borrelia crocidurae. Overall, the work is certainly worthy of investigation, the results are presented well with a few caveats listed below, and the conclusions are justified. My specific comments address items as they appear chronologically in the manuscript, so they are a mixture of minor to more significant issues.

Specific Comments

1. Line 32 (abstract), “relapsed” should be relapsing.

2. Line 37, “Transmitted recurrent fevers (TBRF)” is not correct. TBRF stands for tick-borne relapsing fever, not transmitted recurrent fevers. The authors got it right almost everywhere else, but not here. Also, throughout the authors go back and forth with the full name and the abbreviation. Once tick-borne relapsing fever is abbreviated as TBRF, they should stick with this abbreviation.

3. Line 37-38, the authors state that TBRF are “febrile infections caused by soft ticks of the genus Ornithodoros sp.”. Tick do not cause the febrile infections, spirochetes do. Ticks transmit the spirochetes that cause the infection. This sentence needs to be fixed.

4. Line 45, the authors state “B. crocidurae is recognized as the only vector responsible for (TBRF) disease”. B. crocidurae is not the vector but the causative agent, a spirochete. The authors need to fix this sentence.

5. Line 61-62, change “Ornithodoros sonrai responsible for B. crocidurae” to Ornithodoros sonai responsible for transmitting B. crocidurae”.

6. Line 74-75, the authors state “we used DNA extracted from RTDs to determine the prevalence of TBRF as a cause of fever in RDT-negative patients in Mali.” This is part of the presentation that I found a bit confusing. Were only malaria-negative samples tested, i.e. were all malaria positive samples excluded? See below.

7. Lines 84-86, the authors state that they collected RDT cassettes from 41 sites in 9 administrative units. Because most of the units are large and can vary in their ecology, to me it would be worthwhile to list in a supplementary file what the 41 sites were. Part of the rationale for this is based on their reference 17, which found spirochete infected ticks and small mammals in the Bandiagara region of the Mopti District. Were sites included in this region that included TBRF endemic villages? In the Kayes District, were all (9) positive samples from the same district? If not too difficult, a table giving the sites would be of interest.

8. Lines 133-134, the authors state “A total of 1496 malaria RDT samples (260 positive and 1236 negative from febrile patients were tested for Borrelia spp by real-time PCR.” Here then the authors apparently tested 260 positive malaria samples for Borrelia infection. Thus, this begs the question: were any dual infections found, i.e. patients with both plasmodium and borrelia infections?

9. In the results section, I think the authors should state how much flab sequence they determined and used in their alignments with homologous sequences they obtained from GenBank. The authors provide the nine partial flab sequences in a supplementary file but the reader has to count the bases to determine the amount of sequence used. I did this and the sequences ranged from 322 to 346 bases, with what appears to be good overlap among them. I think the authors should describe these sequences a bit more in the primary text of the article.

10. On a related issue, most journals require novel sequences to be deposited in GenBank with accession numbers for them included in the manuscript. This appears not to have been done. The editors might be best qualified to make this call.

11. Lines 138-140, the authors state “The phylogenetic tree shows that our sequences are close to those already found in Mali, Mauritania and Senegal”. I think the authors should state what the percent identity values are. Also, it might be reasonable to state exactly how much of the partial flaB sequences were compared.

12. Lines 142-143, the authors give in the figure legend “Map of Mali showing the number of malaria RDT cassettes tested in the denominator and the number positive in the numerator.” For clarity, I suggest changing this to “Map of Mali showing the ten administrative districts and the number of malaria RDT cassettes tested in the denominator and the number of Borrelia positive in the numerator.”

13. Line 152, the authors state “Sequences of the B. crocidurae FlaB gene found in O. sonrai ticks”. FlaB should be flaB and the authors should reference the work finding B. crocidurae in these ticks.

14. Lines 157-159, the authors state for reference 17 that “a study conducted in 20 villages in southern Mali reported the presence of the vector O. sonrai in small mammals and ticks collected from rodent burrows”. This is awkward and should be changed to “the presence of the vector O. sonrai in small mammal burrows”. Delete “and ticks collected from rodent”. You don’t find O. sonrai in small mammals.

15. Lines 175-176, the authors state “The increase in the number of cases demonstrates that TBRF is a real public health problem in Senegal [reviewer, I agree] and other neighboring countries such as Mali.” This may be premature to make this claim for Mali given the information presented, nine human infections out of nearly 1500 samples and all restricted to the Kayes district in western Mali. Clearly more work needs to be done.

16. Lines 187-188, the authors state that the results of their study “were similar to those previously found in Senegal [17, 42].” Their reference 17 includes work done in Mali, so Mali should be included at the end of this sentence.

17. Line 325, the reference is incomplete. The journal, volume and pages need to be added.

18. The paper is relatively short, so I think it would be helpful to expand a bit on the methods of how the samples were obtained and what the malaria RDT cassettes provided. I realize the authors cite a paper but one that is not the easiest to access. Did the authors go to major health clinics in all the administrative districts and obtain stored RDT cassettes, sampling 180 at random in each district? What was the time between when the malaria tests were performed and when the blood was eluded and DNA extracted? Were the cassettes held at room temperatures, which can be quite warm in many of the regions sampled. I think a more detailed description of the sampling would be useful and informative.

19. Finally, possibly a table would be quite helpful that breaks down the samples as to how many malaria positive samples, malaria negative samples, borrelia positive samples and borrelia negative samples among them etc were done. Maybe not critical but I think the authors should consider such a table in their results section.

Reviewer #2: 1. Major comments:

According to the submission guidelines of Journal Plos Neglected Tropical Diseases the abstract (with 250-300 words) is conceptually divided into three sections: (i) background, (ii) methodology/Principal findings, (iii) Conclusion/Significance. Please adopt this structuring, and present an author summary before the introduction. The malaria RDTs usually taken for detecting, for example Plasmodium falciparum –extracellular parasite- in febrile patients were been demonstrated as a reliable source of bacterial DNA for the investigation of acute undifferentiated febrile illness or extracellular pathogens from neglected arthropod-borne tropical diseases. This is why they are used in the study to screen for the pathogen bacteria, B. crocidura, in Mali endemic areas. In the manuscript specify anywhere, if necessary, when you must write “malaria RDTs for Plasmodium falciparum“ which includes malaria-positive and malaria negative RDTs for P. falciparum or of other species of malaria such as P. ovale, P. vivax, P. malariae, either “malaria-positive RDTs for P. falciparum (P. f) or malaria-negative RDTs for P. falciparum“ or another malaria species targeted in febrile patients. In the manuscript we do not know the malaria RDTs for which species you collected in health centers in Mali. I imagine you have collected malaria RDTs for P. falciparum which are more commonly used, if both malaria-positive and malaria-negative RDTs P. f were sampled in health centers, please provide as much descriptive information as possible that concerning.

In materials and methods, please replaces the subtitle “study sites“ with “study areas“, so in this subtile where you describe the climatic zones, they should be illustrated with a map representing Figure 1 at line 83 at the end of the sentence. In addition, malaria RDTs were collected in health centers, not in sites, then write rather “malaria RDTs were collected from 41 health centers in 9 regions by indicating at line 84 malaria RDTs for which species targeted (malaria RDTs P. f) you sampled as suggested above with a detailed description of the number of health centers per region where the “malaria RDTs were collected and illustrated with Figure 2, which replace Figure 1, and indicate that you must perform a qPCR P. falciparum to confirm and/or infirm respective positivity or negativity of DNA extracts from malaria-positive and malaria-negative RDTs P. f as false positive and false negative results are sometimes observed in malaria RDTs P. f tested, and also search cases of Borrelia positive DNA extracts from malaria RDTs P. f associated with other pathogens such as P. falciparum, Coxiella burnetii, Bartonella quintana and Rickettsia /Anaplasma spp.

In the results, structure a subtitle such as, (i) “Collection of malaria RDTs for P. falciparum in the targeted health centers“, and describe, with a Table presented, all informations related to health centers targeted per region, geographic coordinates from health centers localities of each region, number of malaria-postive RDTs P. f or malaria-negative RDTs P. f collected and tested, then results of qPCR P. falciparum performed to confirm and/or infirm respective positivity or negativity of DNA extracts from malaria-positive and malaria-negative RDTs P. f, (ii) “Molecular detection of DNA extracts from malaria RDTs for P. falciparum“, and describe all results of DNA extracts from malaria RDTs P. f tested for Borrelia infection both by ITS4 and specific Borcro glpQ qPCR, and of possible cases of co-infections between B. crocidurae / P. falciparum, B. crocidurae / C. burnetii, B. crocidurae / B. quintana, B. crocidurae / Rickettsia /Anaplasma spp. researched.

In the discussion, from line 176 to 182, please remove as this argument does not concern and has no connection with the epidemiological context of TBRF in the endemic areas studied.

In addition, from line 169 to 170 remove inappropriate references [21,39,40] used to explain the impact of climate change, which unfortunately do not correspond to research on climate impacting TBRF, but rather concern Lyme disease and tick-borne encephalitis transmitted by hard species ticks of the genus Ixodes. The only studies known providing data climate influencing TBRF epidemiology were performed by Trape et al., 1996b.

2. Comment of minor points:

a /. Indicate the corresponding author with an asterisk * and mention his email address below the list of authors.

b/. In the abstract, at line 27, replace “sites” with “health centers”.

At line 29 put a comma after the digit in the number 1496 as follows 1,496, then add after the number 9 “DNA extracts from” malaria-negative RDTs…??…., but after RDTs, specify which species of malaria is targeted (malaria RDTs P. f?), “for an overall prevalence of 0.6% (9/1,496)”.

At line 30 to 31, the prevalence rate of B. crocidurae in Kayes (6%, 9/150) is not correct. Please correct with the right ratio 9/180 (5%) and specify after malaria RDTs species of malaria targeted (malaria RDTs P. f?).

At line 34, remove “Malaria” and add “malaria” in front of “RDTs P. f?” then specify species of malaria targeted (malaria RDTs P. f?).

c/. In the introduction, at line 37, the sentence is poorly worded, since Ornithodoros soft ticks are vectors, but not the cause of febrile infections due to B. croicidurae. Then remove the parentheses in TBRF at lines 37, 43 and 45. Rewrite the sentence at lines 37-38 as follows: “Transmitted recurrent fever like TBRF by soft ticks of the genus Ornithodoros are neglected febrile infections due to Borrelia spp.”

At line 44, the reference [9] cited is related to louse-borne disease due to Borrelia recurrentis, please remove.

At line 45, B. crocidurae is not a vector, please reword the sentence.

At line 39, remove “caused by” and replace with “hosting”, then delete “the genus” and add “species” after Borrelia.

Borrelia duttonii and B. recurrentis are not hosted by wild rodents, please remove.

At line 46, it is not true by saying “extreme precarious conditions” for rural populations living in endemic TBRF areas, but rather to rural populations living in traditional dwellings made of mud and sleeping on the floor. The references cited [10,11] not appropriate for TBRF in West Africa, please replace with Trape et al., 1991, 1996, 2013.

At line 61, replace “proliferation” with “spread”, and line 62 replace also “responsible for” with “vector of”.

At line 67, blood smears are a tool (technique) for detecting malaria parasites, please so reword.

From line 71 to 72, add “Malaria” in front of rapid diagnostic tests (RDTs) in the beginning of the sentence and specify malaria species targeted (malaria RDTs for P. falciparum or use P. f?).

Not true to say “due to their easy use” in line 72, remove and write the right reason.

At line 74, it is likely that reference [28] is missing here, please verify. Then replace “DNA extracted from RDTs” with “DNA extracts from RDTs for P. falciparum” to…

At line 75, replace “fever in RDTs-negative” with “acute undifferentiated febrile illness" in patients in Mali.

d/. In materials and methods, replace “RDTs cassettes” with “malaria RDTs for P. falciparum?”, then replace “sites” with “health centers”. You say that malaria RDTs P. f? were collected from June to December 2021 at line 86, while the approval for the use of malaria RDTs (P. f?) has been granted in February 2023, please correct with the right date.

The description of climatic zones must be illustrated with a map showing climatic zones in Mali (title Figure 1 to cite at line 84, end of the sentence), so the Figure 1 becomes Figure 2 to cite at line 87, end of the sentence, a showing the localities where malaria RDTs for P. falciparum? were collected in health centers and results of molecular analysis, in Mali (title Figure 2 at line 142). Please mention “Legend” above Figure 2

Title Figure 1: Map of Mali showing climatic zones (at line 84)

Title Figure 2: Map of Mali showing the localities where malaria RDTs for P. falciparum? were collected in health centers and results of molecular analysis (at line 142), no malaria RDTS P. f collection data on the map, please remove all.

From line 78 to 87, lack of double spacing between lines in this paragraph.

At line 89, add after from “malaria RDTs P. falciparum” performed was ….

At line 110, remove “M” after Rahal, and line 120 the Figure 2 becomes Figure 3.

e/. In ethical considerations, at line 125 replace “RDTs” with “malaria RDTs for P. falciparum?”, and write correctly reference of approval granted under this number 064 at line 129.

f/. In the results, please delete all numbers mentioned in the Figure 2 in each region as these data must be reported in a Table, then indicate legend of screening results. At line 135 Figure 1 becomes Figure 2.

Describe the results of title “Collection of malaria RDTs for P. falciparum in the targeted health centers”, then those of that “Molecular detection of DNA extracts from malaria RDTs for P. falciparum?”.

At line 140, Figure 2 becomes Figure 3. In this Figure 3 replace “Negative RDTs cassettes” with “Malaria-negative RDTs” for P. falciparum, then replace “Host tick” O. sonrai in Senegal Mali and Mauritania with “Vector tick” O. sonrai in Senegal Mali and Mauritania.

At line 150, the Figure 2 becomes Figure 3, then add “malaria-negative RDTs” P. f? after from.

Title Figure 3: Phylogenetic tree (maximum likelihood method, bootsrap 1,000) of partial sequence data for the flagellin gene (FlaB) of B. crocidurae identified from malaria-negative for P. falciparum (at line 150)

g/. In the discussion, at line 157 please provide corresponding reference related to this sentence.

At line 158, add “tick” after vector, and line 159, add “these” after “17.3% of”.

At line 163 to 164, reword the sentence as follows: Our B. crocidurae prevalence found in Kayes (5%, 9/180) close to that of Mediannikov et. al (provide reference) conducted …….

At line 167, after “affected”, remove the colon, and add “with 13.5% of Borrelia infection” (the report 33/173 mentioned is not correct, please provide the right one).

At line 169 to 170, provide appropriate reference.

At line 171, add “Senegal” after “In”, then from line 171 to 175, provide appropriate reference related, end of the sentence.

At line 182, remove “In this context”, and begin the sentence with “A similar study performed by Ndiaye et al.…., then remove “EHI” after Ndiaye at line 183. Add “DNA extracts from malaria RDTs P. falciparum? collected in Senegal” after prevalence of B. crocidurae in”, line 183.

At line 185, after “detected in our”, add “DNA extracts from malaria-negative RDTs P. f? samples was previously been identified”

At line 187, after “the Borrelia” add “detected” in our study

At line 195, after “the disease” add “in endemic rural areas from Mali and West African countries where molecular biology techniques are lacking” (to complete the sentence).

h/. In the references, at line 233 remove reference [9] concerning louse-borne disease.

At line 245 and line 247, reference duplicate, remove one of them [14].

At line 266, 320, 323, remove these inappropriate references [21,39,40].

At line 299 and line 302, reference duplicate, remove one of them [32].

**********

PLOS authors have the option to publish the peer review history of their article (what does this mean?). If published, this will include your full peer review and any attached files.

If you choose “no”, your identity will remain anonymous but your review may still be made public.

**Do you want your identity to be public for this peer review?**  For information about this choice, including consent withdrawal, please see our Privacy Policy.

Reviewer #1: No

Reviewer #2: No

**Figure resubmission:**

**Reproducibility:**

To enhance the reproducibility of your results, we recommend that authors of applicable studies deposit laboratory protocols in protocols.io, where a protocol can be assigned its own identifier (DOI) such that it can be cited independently in the future. Additionally, PLOS ONE offers an option to publish peer-reviewed clinical study protocols. Read more information on sharing protocols at https://plos.org/protocols?utm_medium=editorial-email&utm_source=authorletters&utm_campaign=protocols

Attachment Submitted filename: renamed_88f66.pdf

# Author comment: Author response to Decision Letter 1

**DOI:** 10.1371/journal.pntd.0014033.r002

26 May 2026

Attachment Submitted filename: Reviewers v1.docx

# Editor report: Decision Letter 1

**DOI:** 10.1371/journal.pntd.0014033.r003

**Authors:** Joseph M. Vinetz

10 Jun 2026

Dear Pr Ranque,

We are pleased to inform you that your manuscript 'Quantitative polymerase chain reaction from malaria rapid diagnostic tests to detect Borrelia crocidurae in Mali' has been provisionally accepted for publication in PLOS Neglected Tropical Diseases.

Before your manuscript can be formally accepted you will need to complete some formatting changes, which you will receive in a follow up email. A member of our team will be in touch with a set of requests.

Please note that your manuscript will not be scheduled for publication until you have made the required changes, so a swift response is appreciated.

IMPORTANT: The editorial review process is now complete. PLOS will only permit corrections to spelling, formatting or significant scientific errors from this point onwards. Requests for major changes, or any which affect the scientific understanding of your work, will cause delays to the publication date of your manuscript.

Should you, your institution's press office or the journal office choose to press release your paper, you will automatically be opted out of early publication. We ask that you notify us now if you or your institution is planning to press release the article. All press must be co-ordinated with PLOS.

Thank you again for supporting Open Access publishing; we are looking forward to publishing your work in PLOS Neglected Tropical Diseases.

Best regards,

Joseph M. Vinetz

Section Editor

PLOS Neglected Tropical Diseases

Joseph Vinetz

Section Editor

PLOS Neglected Tropical Diseases

Shaden Kamhawi

co-Editor-in-Chief

PLOS Neglected Tropical Diseases

orcid.org/0000-0003-4304-636XX

Paul Brindley

co-Editor-in-Chief

PLOS Neglected Tropical Diseases

orcid.org/0000-0003-1765-0002

***********************************************************

p.p1 {margin: 0.0px 0.0px 0.0px 0.0px; line-height: 16.0px; font: 14.0px Arial; color: #323333; -webkit-text-stroke: #323333}span.s1 {font-kerning: none

# Editor report: Acceptance letter

**DOI:** 10.1371/journal.pntd.0014033.r004

**Authors:** Joseph M. Vinetz

Dear Pr Ranque,

We are delighted to inform you that your manuscript, "Quantitative polymerase chain reaction from malaria rapid diagnostic tests to detect Borrelia crocidurae in Mali," has been formally accepted for publication in PLOS Neglected Tropical Diseases.

We have now passed your article onto the PLOS Production Department who will complete the rest of the publication process. All authors will receive a confirmation email upon publication.

The corresponding author will soon be receiving a typeset proof for review, to ensure errors have not been introduced during production. Please review the PDF proof of your manuscript carefully, as this is the last chance to correct any scientific or type-setting errors. Please note that major changes, or those which affect the scientific understanding of the work, will likely cause delays to the publication date of your manuscript. Note: Proofs for Front Matter articles (Editorial, Viewpoint, Symposium, Review, etc...) are generated on a different schedule and may not be made available as quickly.

Soon after your final files are uploaded, the early version of your manuscript will be published online unless you opted out of this process. The date of the early version will be your article's publication date. The final article will be published to the same URL, and all versions of the paper will be accessible to readers.

For Research Articles, you will receive an invoice from PLOS for your publication fee after your manuscript has reached the completed accept phase. If you receive an email requesting payment before acceptance or for any other service, this may be a phishing scheme. Learn how to identify phishing emails and protect your accounts at https://explore.plos.org/phishing.

Thank you again for supporting open-access publishing; we are looking forward to publishing your work in PLOS Neglected Tropical Diseases.

Best regards,

Shaden Kamhawi

co-Editor-in-Chief

PLOS Neglected Tropical Diseases

Paul Brindley

co-Editor-in-Chief

PLOS Neglected Tropical Diseases
